# Susceptibility towards Enterotoxigenic *Escherichia coli* F4ac Diarrhea Is Governed by the *MUC13* Gene in Pigs

**DOI:** 10.1371/journal.pone.0044573

**Published:** 2012-09-12

**Authors:** Jun Ren, Xueming Yan, Huashui Ai, Zhiyan Zhang, Xiang Huang, Jing Ouyang, Ming Yang, Huaigu Yang, Pengfei Han, Weihong Zeng, Yijie Chen, Yuanmei Guo, Shijun Xiao, Nengshui Ding, Lusheng Huang

**Affiliations:** 1 Key Laboratory for Animal Biotechnology of Jiangxi Province and the Ministry of Agriculture of China, Jiangxi Agricultural University, Nanchang, People’s Republic of China; 2 College of Life Science, Jiangxi Science and Technology Normal University, Nanchang, People’s Republic of China; National Institute of Allergy and Infectious Diseases, United States of America

## Abstract

Enterotoxigenic *Escherichia coli* (ETEC) F4ac is a major determinant of diarrhea and mortality in neonatal and young pigs. Susceptibility to ETEC F4ac is governed by the intestinal receptor specific for the bacterium and is inherited as a monogenic dominant trait. To identify the receptor gene (F4acR), we first mapped the locus to a 7.8-cM region on pig chromosome 13 using a genome scan with 194 microsatellite markers. A further scan with high density markers on chromosome 13 refined the locus to a 5.7-cM interval. Recombination breakpoint analysis defined the locus within a 2.3-Mb region. Further genome-wide mapping using 39,720 informative SNPs revealed that the most significant markers were proximal to the *MUC13* gene in the 2.3-Mb region. Association studies in a collection of diverse outbred populations strongly supported that *MUC13* is the most likely responsible gene. We characterized the porcine *MUC13* gene that encodes two transcripts: MUC13A and MUC13B. Both transcripts have the characteristic PTS regions of mucins that are enriched in distinct tandem repeats. MUC13B is predicated to be heavily O-glycosylated, forming the binding site of the bacterium; while MUC13A does not have the O-glycosylation binding site. Concordantly, 127 independent pigs homozygous for *MUC13A* across diverse breeds are all resistant to ETEC F4ac, and all 718 susceptible animals from the broad breed panel carry at least one *MUC13B* allele. Altogether, we conclude that susceptibility towards ETEC F4ac is governed by the *MUC13* gene in pigs. The finding has an immediate translation into breeding practice, as it allows us to establish an efficient and accurate diagnostic test for selecting against susceptible animals. Moreover, the finding improves our understanding of mucins that play crucial roles in defense against enteric pathogens. It revealed, for the first time, the direct interaction between MUC13 and enteric bacteria, which is poorly understood in mammals.

## Introduction

Enterotoxigenic *Escherichia coli* (ETEC) expressing the F4 (previously known as K88) fimbriae is a major cause of diarrhea in neonatal and pre-weaned piglets [1], which leads to considerable economical loss in the pig industry. The bacteria use fimbriae to adhere to specific receptors on brush borders of enterocytes of the small intestine. Colonizing bacteria secret the deleterious enterotoxins that cause an increased secretion of electrolytes into the lumen. Subsequently, water flows into the lumen resulting in diarrhea [1].

Three antigenic variants of F4 have been described: F4ab, F4ac and F4ad, of which F4ac is the most prevalent [2]. As early as 1977, Gibbons et al. [3] showed that the adherence to ETEC F4ac was inherited as an autosomal dominant Mendelian trait with the two alleles: *S* (adhesion, dominant) and *s* (non-adhesion, recessive). It is assumed that susceptibility towards ETEC F4ac is determined by the intestinal receptor that allows the bacterium to adhere to the intestinal tract or not. The identification of the receptor locus is thus desirable for the pig industry as it would enable us to accurately and efficiently eliminate the susceptible allele from nucleus breeding populations, leading to decreased mortalities caused by ETEC F4ac infection.

The locus encoding the intestinal receptor for ETEC F4ac, denoted as F4acR, has been initially mapped to the q41 region on pig chromosome 13 (SSC13) by two independent linkage analyses [4]–[5]. The responsible region was subsequently refined to 5.7 cM by a meta-analysis of different experimental populations [6] and narrowed down to an interval of 3.1 Mb by haplotype sharing analysis [7]. More recently, the receptor locus has been further defined within the *LMLN*-*S0283* region by recombination breakpoint analysis [8]. Several interesting candidate genes of F4acR including *MUC4* [9], *MUC13* [10], *MUC20* [11] and *TFRC* [12]–[13] in the critical region have been investigated, and genetic markers significantly associated with *in vitro* F4ac adhesion phenotypes in specific pig populations have been described [9]–[10], [14]–[15]. However, the responsible gene and causal variant(s) of F4acR remains unknown so far. By a battery of genetic analysis, we herein show the compelling evidence that *MUC13* is the responsible gene for the intestinal receptor conferring susceptibility to ETEC F4ac infection in pigs. We further identified *MUC13* markers that are in complete linkage disequilibrium with the resistant causal allele in a broad panel of Western pig populations. The finding allowed us to select for the F4ac resistant animals and would greatly benefit the worldwide pig industry.

## Results and Discussion

### Whole Genome Scan Confirms the Location of F4acR in the q41 Region on SSC13

To identify loci affecting economically important traits in pigs, we constructed a large scale White Duroc × Erhualian F_3_ intercross population [16], in which 755 F_2_ and 461 F_3_ animals were recorded for *in vitro* F4ac adhesion phenotypes by a microscopic enterocyte adhesion assay as described previously [17]. We genotyped the entire F_2_ pedigree for 194 microsatellite markers covering the pig genome and performed a whole genome scan. The linkage analysis mapped F4acR to a region of 7.8 cM flanked by *SW207* and *S0075* in the q41 region on SSC13, which confirmed the previous reports of other investigators [4]–[5].

### Chromosome Scan with High-density Markers on SSC13 Refine F4acR to a 5.7-cM Region

**Figure 1:**
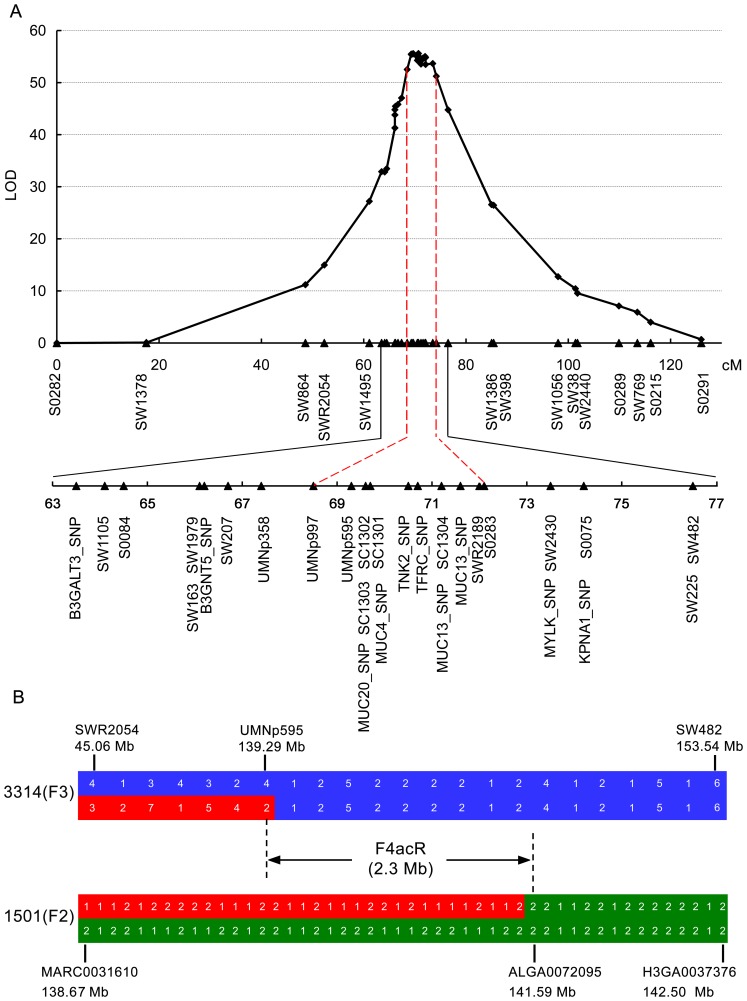
Mapping and fine mapping of the locus for the intestinal F4ac receptor. (A) A chromosome scan with high density markers on pig chromosome 13 mapped the locus to a 5.7-cM region. A total of 50 markers (Table S3) were genotyped on all animals in the White Duroc × Erhualian F_2_ intercross, and a multipoint linkage analysis was performed to localize the receptor locus. The confidence interval from *UMNp997* to *S0283* for the locus is indicated by dashed vertical lines. (B) Recombination breakpoint analyses define the locus within a 2.3-Mb region. The diagram shows recombination breakpoint events in the candidate region of F4acR in individuals 1501 and 3314. The Erhualian-derived resistant chromosome is indicated in blue, and the White Duroc-derived resistant chromosome is marked in green. The recombinant susceptible haplotype from White Duroc founder boars is highlighted in red. Polymorphisms are displayed at the respective gene or microsatellite markers. The positions of polymorphisms (Table S3 and SNPs on the 60k chip) are shown according to the pig genome assembly (Sscrofa10.2). Microsatellite alleles are numbered consecutively from shortest to longest fragments. For SNP markers the allele with the higher frequency is denoted 1, and the allele with the lower frequency is denoted 2.

To refine the location of F4acR, we increased the marker density in the *SW207* - *S0075* interval on SSC13. A panel of 50 informative markers including 32 microsatellite and 18 SNPs on SSC13 were genotyped on all animals in the White Duroc × Erhualian F_2_ cross. A multipoint linkage analysis showed that the *UMNp997*– *S0283* interval of 5.7 cM was defined as the most likely region harboring F4acR as the association of this region was 100-fold stronger than that for any other region in the genome (**Figure 1A**). The result was consistent with the recent mapping report of F4acR by Joller et al. [6].

### Recombination Breakpoint Analysis Defines F4acR within a 2.3-Mb Interval

To further define the physical location of F4acR, we performed recombination breakpoint analysis in the White Duroc × Erhualian F_3_ intercross. The entire cross was genotyped for 23 informative markers flanking the 5.7-cM interval, and the F_2_ pedigree was further genotyped using PorcineSNP60 BeadChips (see below). We identified susceptible and resistant haplotypes of founder animals by their complete association with adhesion and non-adhesion phenotypes in the cross, respectively. Recombination events in the candidate region of F4acR were observed in one F_2_ (individual 1501) and one F_3_ animals (individual 3314). Individual 3314 was a non-adhesive animal and should be homozygous for the resistant allele. In the F4acR region, this animal carried a non-recombinant resistant chromosome from Erhualian founder sows and a recombinant chromosome from White Duroc founder boars. The recombinant *SWR2054*– *UMNp595* interval was identical to the susceptible haplotype, which thus positioned F4acR downstream of *UMNp595* (**Figure 1B**). Individual 1501 showed the adhesive phenotype and should be a carrier of the susceptible allele. The individual carried a non-recombinant resistant chromosome and a recombinant chromosome both from White Duroc founder boars. The recombinant *ALGA0072095* - *H3GA0037376* interval around the F4acR region was a resistant haplotype, which hence mapped F4acR upstream of *ALGA0072095* (**Figure 1B**). Taken together, the breakpoint analysis unambiguously defined F4acR within the *UMNp595*– *ALGA0072095* interval of 2.3 Mb (139.29 Mb –141.59 Mb, Sscrofa10.2) on SSC13 (**Figure 1B**). The responsible region refined the recently described 3.1-Mb interval of F4acR [7].

### Genome-wide Association and Combined Linkage and Linkage Disequilibrium (LDLA) Analyses Reveal *MUC13* as the Most Likely Gene for F4acR

**Figure 2:**
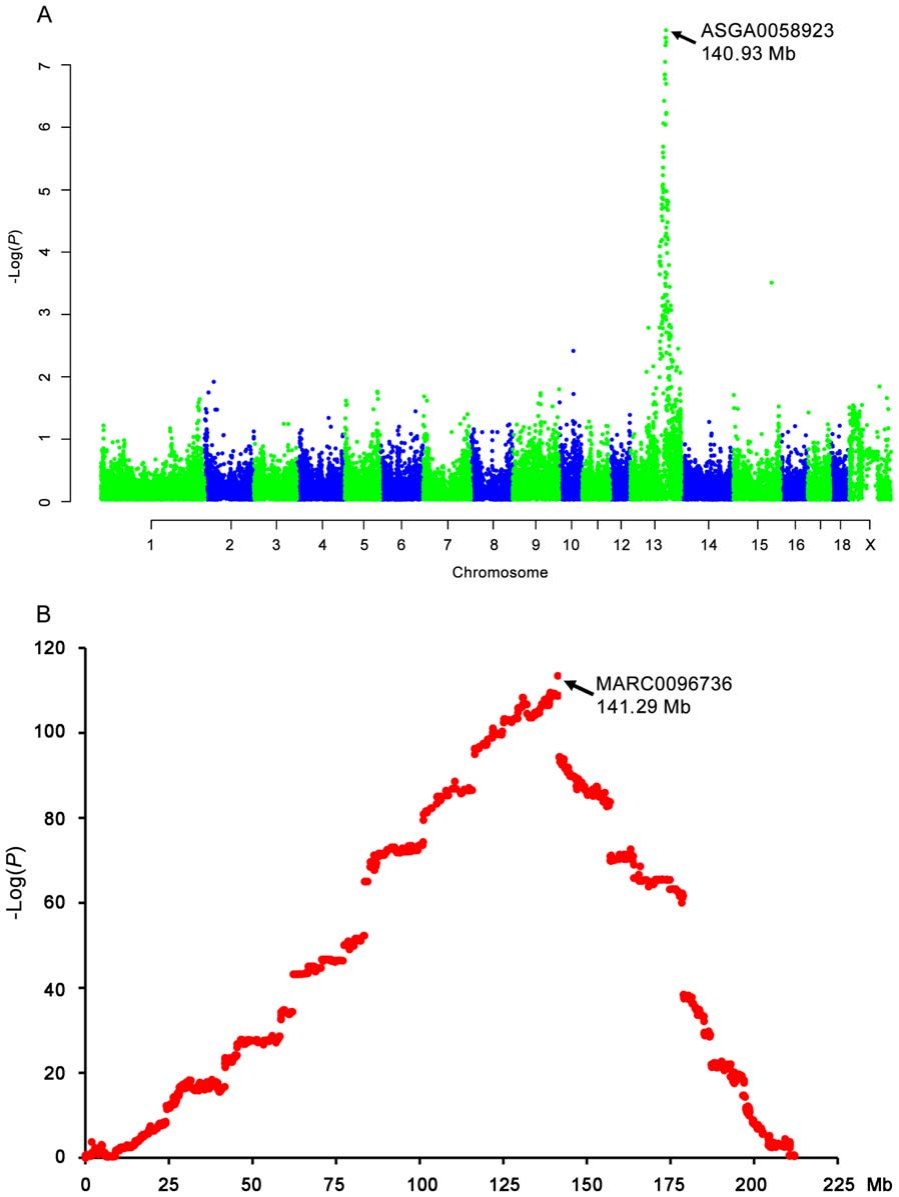
GWAS and LDLA analyses indicate MUC13 as a strong candidate of F4acR. (A) Genome-wide association mapping of the locus in the White Duroc × Erhualian F_2_ intercross using the 60K chips. Evidence for linkage (y axis) is measured as corrected log(1/*p*). The most significantly associated marker is ASGA0058923 at 140.93 Mb (Sscrofa10.2) proximal to the *MUC13* gene (141.02–141.14 Mb, Sscrofa10.2) on chromosome 13. (D) Combined linkage disequilibrium and linkage analysis of the locus in the White Duroc × Erhualian F_2_ intercross using the 60K chips. The graph shows the linkage signal on chromosome 13. The most significant marker is MARC0096736 at 141.29 Mb (Sscrofa10.2) adjacent to the *MUC13* gene.

To pinpoint the most probable candidate gene for F4acR in the defined 2.3-Mb region, we further genotyped all animals across the F_2_ intercross using PorcineSNP60 BeadChips [18]. We performed a genome-wide association study (GWAS) on the basis of 39,720 informative SNPs scan under a dominant model. The GWAS identified the most significantly associated SNP (ASGA0058923, corrected *P* value = 2.98×10^−8^) at 140.93 Mb on SSC13 (Sscrofa10.2, **Figure 2A**). The SNP was located in the 2.3-Mb interval. Six markers in or proximal to the 2.3-Mb region showed similar association strength (*P*<1×10^−7^) as the SNP. We further performed LDLA analysis for F4acR using the 60K chip data and adhesion phenotype data of the F_2_ population. The analysis detected the most significant marker (MARC0096736) in the 2.3-Mb region on SSC13 (**Figure 2B**). The 360-kb interval from 140.93 to 141.29 Mb on SSC13 (Sscrofa10.2) appears to the most probable region of F4acR as it harbors the most significant markers in both GWAS and LDLA assays. The region contains 4 annotated genes: *SLC12A8*, *HEG1*, *ITGB5* and *MUC13*.

Of the 4 genes, *MUC13* appears to be a strong candidate, as F4acR has been shown to be mucin-like sialoglycoproteins [19]–[21]. Mucins form the first line of host defense against enteric pathogens, but are also targets for microbial attachment as they have a variety of oligosaccharide structures providing binding site for bacteria [22]–[23]. MUC13 is a transmembrane mucin that is highly expressed in the jejunum of the pig [10]. It plays a protective role in intestinal inflammation by inhibiting epithelial cell apoptosis in mice [24]. Aberrant expression of human *MUC13* is associated with a variety of epithelial carcinomas, including colorectal, intestinal-type gastric and ovarian cancers (for a review, see [25]). We have previously assumed that *MUC13* is an interesting candidate gene for F4acR [10]. More recently, Fu *et al*. [26] identified five promising candidate genes for F4acR including *MUC13* using GWAS. In the present study, *MUC13* mapped to the 2.3-Mb critical region of F4acR and was proximal to the most significant SNPs in both GWAS and LDLA assays. We thus believe that *MUC13* is the most likely responsible gene for F4acR.

### Association Analysis in Outbred Populations Further Supports *MUC13* as the Responsible Gene of F4acR

To acquire more evidence for the causality of *MUC13*, we characterized a mass of SNP markers around the 2.3-Mb critical region and performed a linkage disequilibrium based association analysis for F4acR in a collection of diverse outbred populations. In detail, we recorded F4ac adhesion phenotypes on 292 unrelated animals from 12 Chinese indigenous breeds and 3 Western commercial breeds (**Table 1**). These animals were genotyped for a total of 188 informative SNPs covering 24 annotated genes in the critical region. Of the 188 SNPs, 79 were from the *MUC13* gene and 53 from another mucin gene (*MUC4*) that has also been proposed as a candidate of F4acR by other investigators [14]–[15]. Given that Chinese and Western pigs have different domestication origin and could differ in causal mutations within the F4acR gene, we first performed association analyses separately on Chinese and Western pigs. We found that *MUC13* g.28784 T>C was the most significantly associated marker in both Chinese and Western pigs. Especially, this SNP had an accuracy of more than 97% (144 out of 148) distinguishing susceptible and resistant animals in the 148 independent Western pigs (**Table 2**). It provides an excellent diagnostic DNA marker for selecting against genetically susceptible animals in Western commercial pigs. We have developed a diagnostic test for the SNP and are applying the test on nucleus animals of Western commercial breeds in China. The result is expected to benefit animals and breeders by protecting against the pathological condition and ensuring economic losses.

**Table 1:** Association of *MUC13A* and *MUC13B* alleles with ETEC F4ac adhesion phenotypes across diverse pig breeds.

	No. of sire family	No. of pig	−/− (*AA*)		−/*del* (*AB*)		*del*/*del* (*BB*)	
Breed			adhesion	non-adhesion	adhesion	non-adhesion	adhesion	non-adhesion
Chinese breeds								
Bamaxiang	6	16	0	5	5	4	1	1
Erhualian	5	14	0	2	2	7	1	2
Hang	3	10	0	6	1	0	3	0
Jiangquhai	3	12	0	0	5	1	4	2
Jinhua	4	11	0	3	0	4	2	2
Laiwu	4	13	0	0	0	0	10	3
Lantang	1	5	0	0	0	1	0	4
Rongchang	4	13	0	0	0	4	0	9
Shaziling	4	8	0	1	2	2	2	1
Sutai	6	166	0	47	17	55	22	25
Tibetan	10	12	0	7	0	4	0	1
Tongcheng	3	10	0	1	5	3	1	0
Yushan Black	3	24	0	2	3	8	3	8
Western breeds								
Duroc	18	46	0	10	13	17	3	3
Landrace	8	32	0	0	4	3	23	2
Large White	21	66	0	5	25	8	28	0
Commercial pigs^a^	24	260	0	35	63	72	48	42
Total	127	718	0	124	145	193	151	105

a Commercial pigs were produced by a three-way cross: Duroc×(Landrace×Large White).

**Table 2:** Genotypes of *MUC13* SNP g.28784 T>C and ETEC F4ac adhesion phenotypes in Western purebred pigs.

Genotype	F4ac adhesion phenotype (n = 144)	
	Adhesion	Non-adhesion
*CC*	0	46
*TC*	60	4
*TT*	34	0

**Figure 3:**
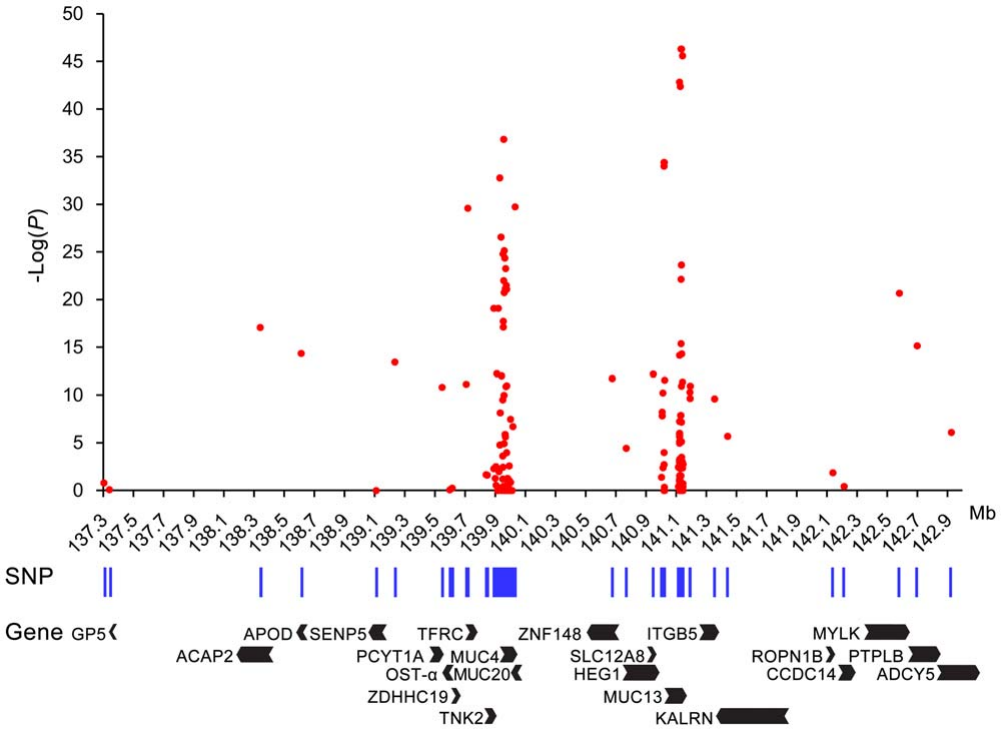
Association of SNPs with ETEC F4ac adhesion phenotypes in outbred populations. In the critical region harboring the receptor locus, 188 informative SNPs were genotyped on 292 independent pigs representing 15 diverse breeds. The positions of SNPs and annotated genes are indicated under the x-axis according to the pig genome assembly (Sscrofa10.2). The associations between SNPs and adhesion phenotypes are presented with *P* values given as –log10P in the y-axis.

Of note, when we performed association analyses across Chinese and Western pigs, the six most significant SNPs were all located in the *MUC13* gene. These SNPs had 1000-fold stronger association than any other SNP including *MUC4* SNPs (**Figure 3**). This observation strengthens the assumption that *MUC13* is the responsible gene for F4acR.

### *MUC13* is a Single Copy Gene that Encodes Two Transcripts (MUC13A and MUC13B) with Distinct PTS Domains

**Figure 4:**
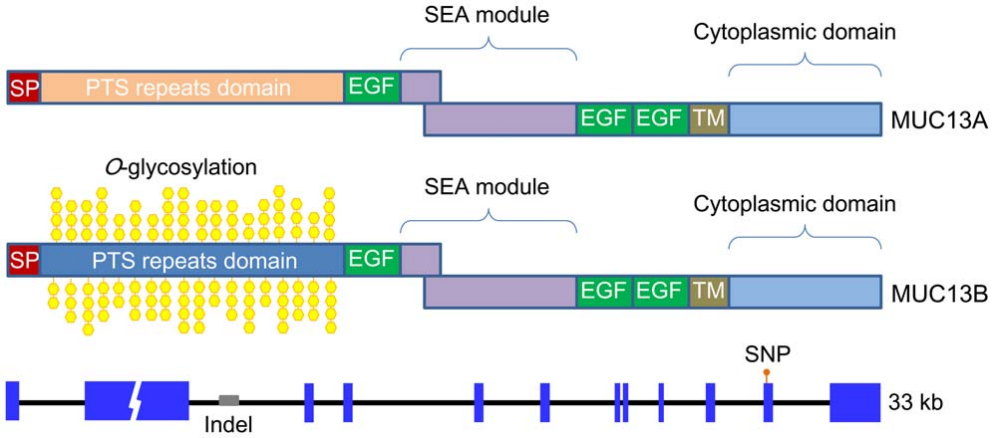
Schematic representation of the predicted protein domains and genomic organization of the porcine *MUC13* gene encoding two transcripts: *MUC13A* (upper panel) and *MUC13B* (middle panel). Exons are indicated by boxes and introns by thin lines. The distinct PTS regions of two *MUC13* transcripts are highlighted by different colors. The gaps on exon 2 indicate the unknown number of tandem repeats in the PTS region that are enriched in proline, threonine and/or serine. We assumed the repeat number as 100 for the analyses of MUC13 PTS domains. The *O*-glycosylated sites are marked only in the PTS region of MUC13B as predicted by DictyOGlyc [45]. The diagnostic Indel for *MUC13A* and *MUC13B* alleles and the most significant SNP (g.28784 T>C) in the association studies of outbred populations are depicted at the corresponding sites. The sizes are drawn to scale.

We have previously isolated a 2679-bp cDNA of pig *MUC13* (NM_001105293) that was highly expressed in the jejunum. As the deduced MUC13 protein lacked the typical PTS region of mammalian mucins in the N-terminus that is enriched in proline, threonine and/or serine, we speculated that pig *MUC13* could have another much longer transcript containing the PTS region [10]. To test this hypothesis, we performed rapid amplification of 5′cDNA end (5′RACE) assays using both Clontech SMART and TaKaRa technologies as described in Method. The RACE assays identified two extended *MUC13* transcripts compared with our previous finding [10]. The two transcripts, namely *MUC13A* (JN613414) and *MUC13B* (JN613417), share the same 5′UTR of 35 bp, transcription start site and 3′UTR of 1497 bp, but have distinct PTS regions that are rich in tandem repeats spanning approximate 3–5 kb (**Figure 4**). The PTS oligopeptide core repeat units in MUC13A are 8–9 amino acid residues with the two most abundant types of ASTSAPSA and ASTSAPAAG; while the repeat unit in *MUC13B* is a string of 8 amino acid residues comprising threonine and proline (TPTPTTTP or TPTPTTTL). It is noteworthy that we failed to characterize the exact number and length of repeats of both transcripts, as the repetitive sequences were unsuccessfully amplified or sequenced using the current available technologies possibly due to the complex second structures of the sequences. Nevertheless, Southern blot analysis revealed that the length of the tandem repeat region was approximate 3–5 kb long (data not shown).

To determine the complete genomic DNA sequence of pig *MUC13*, we screened 4 pig genomic DNA libraries and identified positive BAC/PAC clones encompassing the *MUC13* gene from the libraries. By using the Solexa deep sequencing technology, we obtained the DNA sequences of these clones (JN613413, JN613416) and characterized the genomic structure of the porcine *MUC13* gene. Each BAC clone contained a single *MUC13* gene, corresponding to one of the above-mentioned two transcripts of *MUC13* (**Figure 4**). The two types of *MUC13* DNA sequences (JN613415, JN613418) exhibit a high degree of sequence identity at the nucleotide level (>95%), and both consist of 12 exons and 11 introns (**Figure 4**). The different nucleotides between *MUC13A* and *MUC13B* DNA sequences are predominantly presented in the PTS region on exon 2 and its flanking intronic sequences. For instance, we identified an Indel of 68 bp in intron 2 with the longer sequence for *MUC13A* and the shortened sequence for *MUC13B* (**Figure S1**). The Indel was used as a diagnostic marker for *MUC13A* and *MUC13B* alleles for the following analysis. Like the cDNA analysis, we unsuccessfully determined the complete DNA sequence of the PTS regions as the Solexa sequencing technology generated short pair-end reads of 148 bp that can not reveal the definite number of tandem repeats. Sequencing mucin genes has been shown to be technically difficult due to the large size and the repetitive structure of these molecules. For example, the missing sequence information for the PTS region is also encountered for *MUC3A*, *MUC6*, *MUC7*, *MUC12* and *MUC13* in cattle [27].

**Figure 5:**
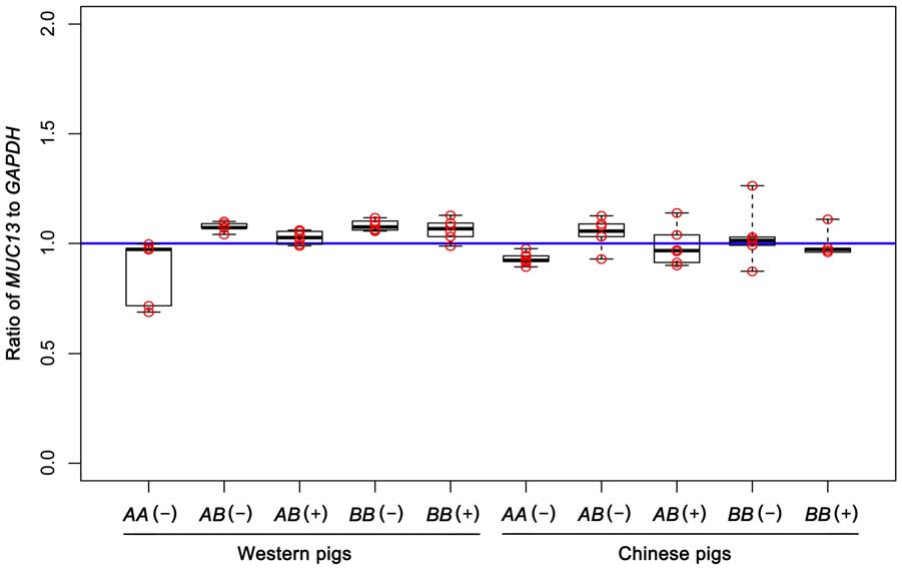
The quantitative analysis for *MUC13* copies by a real time TaqMan PCR assay. Both susceptible (+) and resistant (−) animals randomly sampled from Western and Chinese pigs were used for the copy number assay. These animals (n = 60) were classified into 10 groups according to their genotypes at the diagnostic Indel site and F4ac adhesion phenotypes. Each group included 6 animals, and each animal were analyzed in triplicate. Estimation of copy number was performed by the comparative CT relative quantification assay. The y-axis is the ratio of *MUC13* copy to the reference *GAPDH* copy. The assay shows that the porcine *MUC13* gene is a single copy gene.

To examine if *MUC13A* and *MUC13B* transcripts are encoded by a single gene or two separate loci, we developed a genomic qPCR assay to quantify copy numbers of *MUC13* in the pig genome. The copy number assay measured the relative copy ratio between *MUC13* and the reference *GAPDH* gene. We performed the assay on 60 representative pigs from Chinese and Western diverse breeds. The assay showed that all tested animals had a single *MUC13* gene with the copy number ratio of 1.0 to *GAPDH* **(** **Figure 5**). It demonstrates that *MUC13A* and *MUC13B* transcripts are encoded by a single *MUC13* gene in the pig genome.

### *MUC13A* is Completely Associated with the Resistant Phenotype Across Diverse Breeds

To examine the effect of *MUC13A* and *MUC13B* alleles on susceptibility to ETEC F4ac, we genotyped a large sample of pigs (n = 718) from diverse breeds for the diagnostic Indel marker, and analyzed association of the two *MUC13* alleles with F4ac adhesion phenotypes in these pigs. We found that all 124 pigs homozygous for the *MUC13A* allele from the broad breed panel were resistant (non-adhesive) to ETEC F4ac. Moreover, all 594 susceptible animals carried at least one *MUC13B* allele (**Table 1**). The complete association of *MUC13A* with the non-adhesive phenotype across diverse breeds provides compelling evidence that resistance to ETEC F4ac is governed by the porcine *MUC13* gene. It is noteworthy that the *MUC13B* allele is associated with both susceptibility and resistance towards ETEC F4ac. Nevertheless, we noticed that of the 188 SNPs around the 2.3-Mb region, only *MUC13* SNPs (n = 10) showed the complete (100%) association with the adhesion phenotypes in Western *MUC13B* homozygous pigs (**Table S1**). It further supported the *MUC13* gene as F4acR.

### MUC13 is Perfectly Concordant with the Biochemical Prosperity of F4acR

To elucidate why *MUC13B* is associated with both susceptibility and resistance while *MUC13A* confers only resistance to ETEC F4ac, we analyzed the *O*-glycosylated site of the two *MUC13* transcripts as the site is presumed to be the binding site of the bacteria [23]. It has been well shown that mucins are often very densely *O*-glycosylated, i.e. addition of many short *O*-linked glycans, such as N-acetyl-galatosamine (GalNAc), to the peptides of mucins. The *O*-glycosylation is essential for the function of mucins as it is required to maintain an extended conformation to create a long, filamentous structure. The highly elaborate structures allow mucins to mediate the interactions between epithelia and their surroundings. The abnormal interactions have been implicated in many disease processes including infectious and inflammatory diseases, cancer and metastasis [23].

Protein motif/domain analysis showed that MUC13A does not have *O*-glycosylation sites (**Figure 4** **,** **Figure S2**). This indicates that the peptide of MUC13A can not form the proper filamentous structure by the *O*-glycosylation for the attachment of ETEC F4 fimbriae. It hence explains why *MUC13A* homozygous animals are all resistant to ETEC F4ac. For MUC13B, it has potential *O*-glycosylation sites predominantly in the PTS region (**Figure 4** **,** **Figure S2**). Therefore, MUC13B could be heavily or lightly *O*-glycosylated depending on the variable tandem repeat sequences of the PTS region. This is concordant with the observation that *MUC13B* is associated with both susceptibility and resistance towards ETEC F4ac.

It has been reported that the intestinal receptor for F4ac is *O*-linked mucin-type sialoglycoproteins of 210–240 kDa [19]–[21]. The most abundant amino acids for the receptor proteins are threonine (49%) and proline (25%) [19]. The PTS region of MUC13B is enriched in tandem repeats of TPTPTTTP or TPTPTTTL with a proportion of threonine to proline being about 2∶1, which is perfectly consistent with the protein properties of F4ac receptor. A search of homologous sequence against the latest porcine genome assembly (Sscrofa10.2) did not find any other sequence similar to the *MUC13B* PTS sequence. These findings give additional strong supporting evidence for the *MUC13* gene determining susceptibility/resistance to ETEC F4ac.

### Variable Tandem Repeats (VNTR) in the PTS Region of *MUC13* are Potential Causal Variant(s)

Quantitative RT-PCR analysis showed that the expression level of *MUC13* did not differ significantly in the small intestine of susceptible and resistant animals (**Figure S3**). The finding is consistent with the recent report that the expression of *MUC13* is not related to susceptibility towards ETEC F4ac [28]. This indicates that the more probable causative mutation(s) are coding variants altering the function of MUC13. To identify *MUC13* causative mutation(s), we screened variants in the complete coding region except for the PTS repetitive sequences using RNA of susceptible and resistant animals from both White Duroc and Erhualian breeds. We detected 14 nonsynonymous mutations out of 24 cSNPs. All cSNPs along with 55 intronic SNPs of *MUC13* were included in the data set of 188 SNPs that were genotyped on the 292 outbred pigs. To test if these mutations of interest contribute to susceptibility towards ETEC F4ac, we analyzed their association with the adhesion phenotypes in the 292 animals. The protein-altered SNPs occurred in both susceptible and resistance animals, thereby excluding them as the causative mutation.

As mentioned above, the large PTS regions with variable tandem repeat sequences are characteristics of mucins. The regions constitute *O*-glycolysation sites that are essential for the biological functions of mucins. Hence, variance in the number, length and sequence of the tandem repeats can impact the extent and type of glycosylation and consequently the functions of mucins. For example, the variable tandem repeats in a variety of mucins have been associated with disease susceptibility in humans (for a review, see [29]). This knowledge led us to hypothesize that variable tandem repeats in the PTS region are the most probable causative mutations in the *MUC13* gene. Chinese and Western pigs are expected to have evolved multiple VNTR alleles in the PTS region that govern susceptibility/resistance to ETEC F4ac. If so, it is unlikely to detect SNPs showing complete LD with the causative mutations, just as observed in this study. Currently, the variable tandem repeats can not be characterized due to amplification and sequencing failure. Further investigation will be directed to validate our hypothesis using the next-generation technologies.

### Summary of the Supporting Evidence for *MUC13* as the Responsible Gene

We herein described the causality of the *MUC13* gene for susceptibility/resistance to ETEC F4ac in pigs. The causality is established on the basis of the following arguments: (1) *MUC13* maps to the 2.3-Mb critical region containing F4acR; (2) *MUC13* is proximal to the most significant markers in both GWAS and LDLA analyses based on large scale SNPs scan across the pig genome; (3) Of the 188 SNPs around the critical region, the six most significant SNPs that had 1000-fold stronger association than any other SNP in diverse outbred populations were all located in *MUC13* (4) *MUC13A* allele was completely associated with the F4ac non-adhesion phenotype across diverse pig populations; (5) All susceptible animals from the broad breed panel carried at least one *MUC13B* allele; (6) MUC13 is perfectly consistent with the known biochemical properties of F4acR, as MUC13B has the unique *O*-glycosylation region that forms the binding site of bacterium and is rich in threonine and proline while MUC13A does not. Altogether, these data allow us to conclude that the *MUC13* gene confer susceptibility/resistance to ETEC F4ac in pigs.

Overall, our findings have important practical consequences and will have immediate impact on pig breeding programs, as they allow the rapid elimination of the susceptible allele and consequently greatly benefit animal welfare and the pig industry. The findings also provide novel insights into the functions of mammalian mucins, as it establishes, for the first time, the direct interaction between MUC13 and enteric bacteria. Further endeavors will be directed to identify causative mutations in the *MUC13* PTS region that can not be amplified and sequenced using the current technologies.

## Methods

### Ethics Statement

All animal work was conducted according to the guidelines for the care and use of experimental animals established by the Ministry of Agriculture of China. The ethics committee of Jiangxi Agricultural University specifically approved this study.

### Animals

Experimental animals were from one White Duroc × Erhualian F_3_ intercross population, one Western commercial population, one Chinese cultivated population (Sutai) and 15 outbred populations. The intercross population was constructed with two divergent founder breeds: White Duroc and Chinese Erhualian. Two White Duroc boars were mated to 17 Erhualian sows, and 9 F_1_ boars were then intercrossed with 59 F_1_ sows avoiding full-sib mating to generate 1912 F_2_ animals, of which 87 boars and 299 sows were intercrossed to produce 5311 F_3_ animals. In this study, 755 F_2_ and 461 F_3_ animals at day 240 were slaughtered for ETEC F4ac adhesion phenotype recording. The management of the experimental population has been described previously [16]. The Western commercial population included 260 hybrid pigs at day 180 that were produced from a three-way cross between 24 Duroc boars and 24 Landrace × Large White hybrid sows in 5 farms. The Chinese Sutai population comprised 166 adult pigs at day 240 from 6 sire families. The breed was developed after 18-genereation selection from a Duroc (50%) × Erhualian (50%) cross in 1986. A total of 292 unrelated individuals at the age of 6 to 8 weeks were sampled from 15 Chinese breeds and 3 Western breeds (**Table 1**). To represent a broad consanguinityhttp://www.iciba.com/strain/, animals of each Chinese breed except for Lantang pigs were collected from at least 3 unrelated sire families (no common ancestry for 3 generations) each with 2 to 4 animals. For the three Western breeds, piglets of each breed were collected from 5 nucleus populations representing 18 (Duroc), 8 (Landrace) and 21 (Large White) sire families. Genomic DNA was extracted from ear tissues using a routine phenol/chloroform way and diluted to a final concentration of 20 ng/µl.

### Phenotype Recording

A microscopic enterocyte adhesion assay developed by Baker et al. [30] was adopted to record *in vitro* ETEC F4ac adhesion phenotypes with slight modification as described previously [17]. In brief, brush borders of enterocytes were harvested from a 2-cm segment of the jejunum collected from each animal within 30 min after slaughter. These brush borders were then incubated with F4ac bacterial suspension and 50 µl of mannose (0.4 mg ml^−1^) at 37°C for 30 min with gentle shaking. Each brush border was subsequently tested for its adhesion with F4ac by a phase contrast microscopy (Leica). A total of 20 well-separated and intact brush borders were examined in each specimen. In cases where less than four brush borders bound more than two bacteria, an additional 20 brush borders were scored. According to the classification standard proposed by Baker *et al*. [30], individuals were classified as susceptible (adhesive) to ETEC F4ac when at least 10% of the brush borders bound more than two bacteria. Specimens with all brush borders bound by less than two bacteria were considered as resistant (non-adhesive) subjects. Otherwise they were considered as weakly adhesive animals.

### Whole Genome and Chromosome Scan

A panel of 194 informative microsatellite markers covering the pig genome was genotyped across the White Duroc × Erhualian F_2_ population as described in Guo et al. [16]. To identify SNPs in the mapped region of F4acR on SSC13, genomic DNA of F_1_ boars was amplified with primers listed in **Table S2** and amplicons were directly sequenced in a 3130xl Genetic Analyzer (Applied Biosystem) using original primers. Additional microsatellite markers in the critical region were mined from the pig genome assembly (Sscrofa10 at http://www.ncbi.nlm.nih.gov/mapview/map_search.cgi?taxid=9823). The newly developed microsatellite and SNP markers were genotyped for all animals in the intercross F_2_ pedigree with primers given in **Table S3** by using the fluoresce dye labeled primers (for microsatellite), SNapshot (Applied Biosystem) and PCR-RFLP technologies. A multipoint linkage analysis was performed on SSC13 with Allegro version 2 [31].

### Recombination Breakpoint Analysis

Haplotypes in the candidate region of F4acR were reconstructed for all tested animals in the White Duroc × Erhualian cross using SimWalk2 software [32]–[33]. Susceptible and resistant haplotypes were determined by their complete association with adhesion (susceptible) and non-adhesion (resistant) phenotypes in the resource population, respectively. F_2_ and F_3_ animals that carried a recombinant susceptible haplotype from the founder animals in the F4acR region were explored to define the genomic location of F4acR by recombination breakpoints.

### Genome-wide Association and LDLA Analyses

The PorcineSNP60 BeadChips (Illumina) were used to genotype all animals across the White Duroc × Erhualian F_2_ intercross on an Illumina iScan System following the manufacture’s protocol. The bead arrays with call rate <85% were excluded for further analyses. Genome-wide association studies were performed on all SNPs with a minor allele frequency (MAF) >0.05 and call rate >95% by GenABEL [34]. First, a generalized linear mixed model was performed to adjust polygenic effect. The model was formulated as: y = u+Zu’+e, where y is the adhesion phenotypes (1 for adhesive and 0 for non-adhesive), u is the mean, Z is the kinship matrix and u’ is the random effect. The residual from the fitted model was then used to evaluate association with genotypes by a score test [35]–[36].

LDLA was performed in a haplotype-based approach with the assumption that each current founder population was originated from a history population (K = 20) after recombination and drift of N generations. The haplotypes of the history population can be reconstructed by a Hidden Marcov model [37]. For each individual in the intercross population, we can trace back its genotype at each polymorphic site to its ancenstor K. Association of the adhesion phenotypes (1 for adhesive and 0 for non-adhesive) with genotypes were finally tested.

### Association of SNPs with F4ac Adhesion Phenotypes in Outbred Populations

Polymorphisms in the responsible region of F4acR defined by recombinant breakpoint analysis were identified by comparative sequencing of genomic DNA of two adhesive White Duroc and two resistant Chinese Erhualian animals using primers given in **Table S4**. A final panel of 188 informative SNP markers (**Table S4**) in 24 genes was genotyped on the 292 purebred animals with the adhesion phenotypes by iPLEX SEQUENOM MassARRAY platform. SNP genotype calls were filtered and checked manually, and aggressive calls were omitted from the dataset. Associations of SNP markers with F4ac adhesion phenotypes were evaluated with the standard χ-test implanted in BEAGLE [38].

### Isolation of the Complete cDNA and Genomic DNA Sequence of the *MUC13* Gene

*MUC13* specific primers F1/R1 and F2/R2 (**Table S5**) were designed from the 5′- and 3′- regions of the previously isolated *MUC13* mRNA sequence (NM_001105293). Three BAC and one PAC clones harboring the complete porcine *MUC13* gene were identified by PCR screening of 4 genomic DNA libraries constructed from Western [39]–[41] or Chinese Erhualian pigs [42] using the *MUC13* specific primers. These clones were sequenced at 300×coverage by the Solexa (Illumina) technology at Beijing Genomic Institute, Shenzhen.

Total RNA was extracted from the jejunum of both adhesive and non-adhesive F_2_ animals using the Rneasy Fibrous Tissue Mini Kit (Qiagen). The first strand-complementary DNA was synthesized with the SMART RACE cDNA synthesis Kit (Clontech) and the 5′-Full RACE Kit (TaKaRa). To obtain the extended 5′-end of *MUC13* cDNA, the first strand cDNA (Clontech) was first amplified with *MUC13* nested primers F3/NF3 (**Table S5**) and universal primers UPM/NUP (Clontech, **Table S5**). To isolate the further 5′-end sequence of cDNA, primers F4/NF4 and F5/NF5 (**Table S5**) were designed from a conserved region of the first exon of mammalian *MUC13* and the extended 5′ cDNA sequence by Clontech RACE. The primers together with 5′RACE Outer and Inner Primers (TaKaRa, **Table S5**) were used to amplify the first strand cDNA (TaKaRa). Primers F6/R6 (**Table S5**) were designed to amplify a fragment filling the gap of *MUC13A* transcript. All RACE PCR products were cloned to pGEM-T Easy Vector (Promega) for sequencing analysis using M13 universal primer. The full-length *MUC13* cDNA sequence was obtained by joining the 5′RACE amplicon sequences with the previously isolated cDNA sequence [10]. The complete *MUC13* genomic DNA sequence was determined by the alignment of the obtained cDNA sequence with the BAC/PAC sequences.

### Copy Number Assay

*MUC13* and *GAPDH* specific amplicons of 437 bp and 368 bp were generated by routine PCR with primers MUC13-FP1/RP1 and GAPDH-FP1/RP1 (**Table S6**), respectively. The two amplicons were connected to form an 805-bp fragment by bridge PCR using primers MUC13-FP1 and GAPDH-RP1 (**Table S6**). The fused fragment was cloned into a pGEM-T Easy vector (Promega). Sequence analysis confirmed that the recombinant plasmid clone contained a single copy of *MUC13* and *GAPDH* fragments. The plasmid DNA was used as the reference sample in subsequent genomic qPCR assays, which determined copy numbers of *MUC13* in the pig genome.

TaqMan probes and primers (**Table S6**) were designed for target (*MUC13*) and reference (*GAPDH*) genes. The target and reference probes were 5′ labeled with 6-FAM and VIC, respectively. Both probes were 3′ labeled with the minor groove binder non-fluorescent quencher (ABI). The amplification efficiencies of *MUC13* and *GAPDH* were measured and validated by the *C*_T_ slope method over a fivefold range dilution of the reference DNA. Standard curves were created by plotting the *C*_T_ values against the logarithm amount of DNA. Genomic qPCR assays were performed using 60 independent animals from Chinese and Western diverse breeds. The 60 animals were classified into 10 groups according to their adhesion phenotypes and genotypes at *MUC13A* and *MUC13B* alleles. The target/reference ratios of all samples are normalized by the target/reference ratio of the calibrator sample (the plasmid DNA) using the method described in [43]. Each sample was analyzed in triplicate. The results are expressed as a fold ratio of the normalized target amounts to the reference amounts. All quantitative PCR were performed on a 7500 FAST Real-Time PCR system (ABI).

### Genotyping of *MUC13* Polymorphisms

The Indel in intron 2 distinguishing *MUC13A* and *MUC13B* alleles was genotyped by direct amplification using primers F7/R7 (F7: 5′-TTC TAC TCT GAT TCC ACA TCA CG-3′; R7: 5′-TGG TCA TGT CTA GGA CTC TTT GAG-3′). The *MUC13A* allele was indicated by amplicons of 151 bp, and the *MUC13B* allele was represented by amplicons of 83 bp. The diagnostic test for the most significant *MUC13* marker (g.28784 T>C) in outbred populations was performed using the ABI SNapshot protocol. A 280-bp DNA fragment was amplified with the F8/R8 primer pairs (F8: 5′-GGA GAG ACC AAA CCC ACA GA-3′; R8: 5′-CTC CTC ACC AGC TCC TTA GC-3′). SNapshot reactions were performed with Multiplex Ready Reaction Mix (Applied Biosystem) and an extension primer (5′-TTT TTT TTT TTT TTT CCA TGT ACA TTT CAG AGT CTG AGG GAT-3′) using an ABI 3130XL Genetic Analyzer (Applied Biosystem).

### Computational Analyses of MUC13 Domains

Computational analyses were performed to identify the protein domains of MUC13A and MUC13B. Since the exact number of tandem repeat in the PTS region was not known, we initially assumed this repeat number as 10 for the following analyses. To make sure the analyses to be robust, we compared the results from the sequences with repeat number varying from 10 to 100. The protein domains were identified using Pfam [44]; the GlcNAc *O*-glycosylation sites and N-Glycosylation sites were predicted using the DictyOGlyc and NetNGlyc server, respectively [45]; the coil-coiled structures were analyzed using COILS [46]; and the disorder regions were predicted using RONN [47].

## Supporting Information

Figure S1 **Detection of the diagnostic Indel marker for** ***MUC13A*** **and** ***MUC13B*** **alleles by PCR analysis.** Genomic DNA was amplified with forward (5′-TTC TAC TCT GAT TCC ACA TCA CG-3′) and reverse (5′-TGG TCA TGT CTA GGA CTC TTT GAG-3′) primers. Amplicons of 151 bp and 83 bp indicate the *MUC13A* and *MUC13B* alleles, respectively. Lanes 1–3, 5, 8, 11 and 12: *AB*; lane 10: *AA*; lanes 4, 6, 7 and 9: *BB*; M: 50 bp marker.
(TIF)

Figure S2 **Plots of probabilities indicating the potential** ***O*** **-glycosylation sites in the deduced peptides of MUC13A (upper panel) and MUC13B (lower panel).** The positions of amino acids are given on the x-axis. Vertical green lines indicate the probabilities for the *O*-glycosylation at each residue. The red line indicates the threshold for the predicted *O*-glycosylation site.
(TIF)

Figure S3 **Real-time RT-PCR analysis of** ***MUC13B*** **expression in the small intestine of susceptible and resistant animals from White Duroc and Erhualian breeds.** Tissue samples were collected from piglets at the age of 6–8 weeks for RNA extraction. Three susceptible and three resistant animals homozygous for *MUC13B* were sampled from each breed. Real-time PCR was performed in triplicate. *MUC13B* expression levels normalized with *β-actin* are given (mean ± s.e.). No significant difference was observed in *MUC13B* expression levels between susceptible and resistant pigs. EHL+: Erhualian adhesive pigs; EHL-: Erhualian non-adhesive pigs; WD+: White Duroc adhesive pigs; WD-: White Duroc non-adhesive pigs.
(TIF)

Table S1 **The complete association of** ***MUC13B*** **SNPs with F4ac adhesion phenotypes in Western purebred pigs.**
(DOC)

Table S2 **Primers for identification of SNP markers in the region of F4acR that were genotyped in the intercross population.**
(DOC)

Table S3 **The microsatellite and SNP markers in the region of F4acR that were genotyped in the intercross population.**
(DOC)

Table S4 **Primers for identification of SNP markers in the region of F4acR that were genotyped in outbred populations.**
(DOC)

Table S5 **Primers for isolation of the full-length cDNA and genomic DNA sequence of the porcine** ***MUC13*** **gene.**
(DOC)

Table S6 **Primers for the copy number assay of the porcine** ***MUC13*** **gene.**
(DOC)
