# Supplementary figures and images for: Susceptibility towards Enterotoxigenic Escherichia coli F4ac Diarrhea Is Governed by the MUC13 Gene in Pigs
Source: PLoS One. 2012 Sep 12;7(9):e44573. doi: 10.1371/journal.pone.0044573 (PMC3440394; doi:10.1371/journal.pone.0044573)

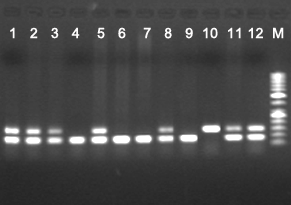

Supplement: Figure S1 — Detection of the diagnostic Indel marker for MUC13A and MUC13B alleles by PCR analysis. Genomic DNA was amplified with forward (5′-TTC TAC TCT GAT TCC ACA TCA CG-3′) and reverse (5′-TGG TCA TGT CTA GGA CTC TTT GAG-3′) primers. Amplicons of 151 bp and 83 bp indicate the MUC13A and MUC13B alleles, respectively. Lanes 1–3, 5, 8, 11 and 12: AB; lane 10: AA; lanes 4, 6, 7 and 9: BB; M: 50 bp marker. (TIF) [file pone.0044573.s001.tif]

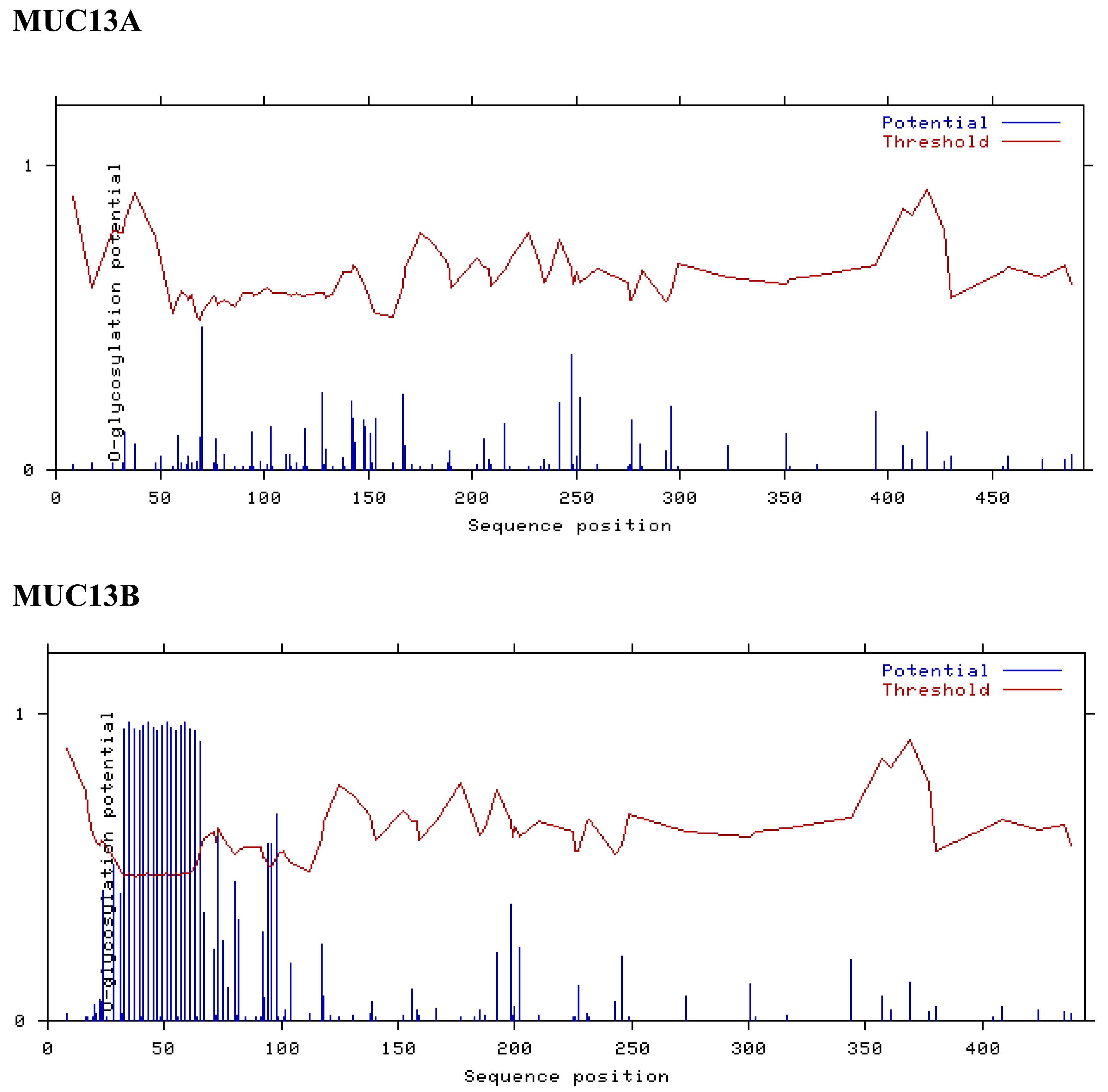

Supplement: Figure S2 — Plots of probabilities indicating the potential O -glycosylation sites in the deduced peptides of MUC13A (upper panel) and MUC13B (lower panel). The positions of amino acids are given on the x-axis. Vertical green lines indicate the probabilities for the O-glycosylation at each residue. The red line indicates the threshold for the predicted O-glycosylation site. (TIF) [file pone.0044573.s002.tif]

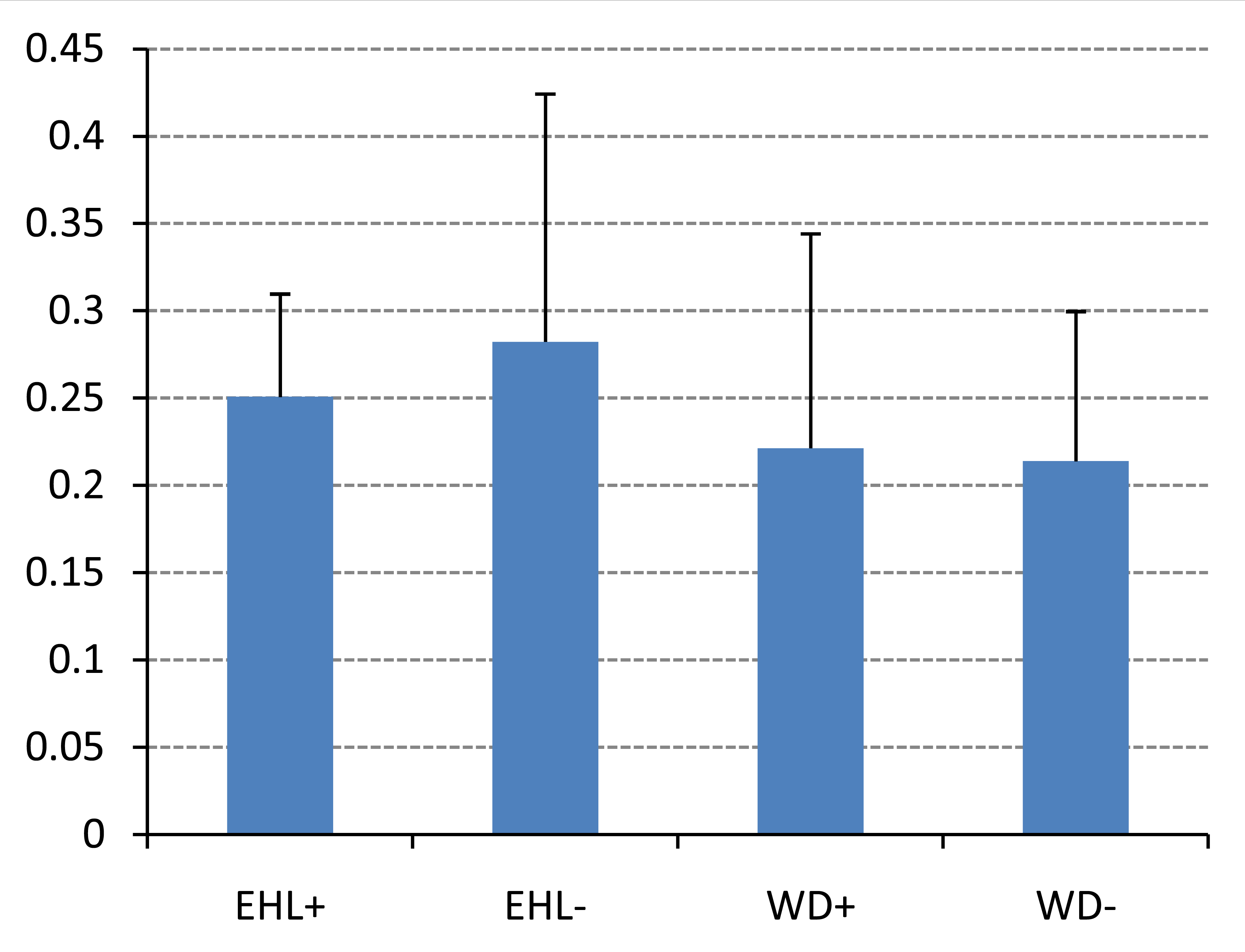

Supplement: Figure S3 — Real-time RT-PCR analysis of MUC13B expression in the small intestine of susceptible and resistant animals from White Duroc and Erhualian breeds. Tissue samples were collected from piglets at the age of 6–8 weeks for RNA extraction. Three susceptible and three resistant animals homozygous for MUC13B were sampled from each breed. Real-time PCR was performed in triplicate. MUC13B expression levels normalized with β-actin are given (mean ± s.e.). No significant difference was observed in MUC13B expression levels between susceptible and resistant pigs. EHL+: Erhualian adhesive pigs; EHL-: Erhualian non-adhesive pigs; WD+: White Duroc adhesive pigs; WD-: White Duroc non-adhesive pigs. (TIF) [file pone.0044573.s003.tif]
