# Supplementary material for: Susceptibility towards Enterotoxigenic Escherichia coli F4ac Diarrhea Is Governed by the MUC13 Gene in Pigs
Source: PLoS One. 2012 Sep 12;7(9):e44573. doi: 10.1371/journal.pone.0044573 (PMC3440394; doi:10.1371/journal.pone.0044573)
Supplement: Table S1 — The complete association of MUC13B SNPs with F4ac adhesion phenotypes in Western purebred pigs. (DOC) [file pone.0044573.s004.doc]

**Supplementary Table 1 The complete association of *MUC13B* SNPs with F4ac adhesion phenotypes in Western purebred pigs.**

| SNP | Genotype | F4ac adhesion phenotype | |
| --- | --- | --- | --- |
|  |  | Adhesion | Non-adhesion |
| g.18553 A>C | *AA* | 34 | 0 |
|  | *AC* | 20 | 0 |
|  | *CC* | 0 | 5 |
| g.18957 C>A | *AA* | 0 | 5 |
|  | *CA* | 18 | 0 |
|  | *CC* | 36 | 0 |
| g.19119 G>A | *AA* | 0 | 5 |
|  | *GA* | 18 | 0 |
|  | *GG* | 36 | 0 |
| g.21471 C>T | *TT* | 0 | 5 |
|  | *CT* | 20 | 0 |
|  | *CC* | 34 | 0 |
| g.21704 G>A | *GG* | 34 | 0 |
|  | *GA* | 20 | 0 |
|  | *AA* | 0 | 5 |
| g.22124 T>C^a^ | *CC* | 0 | 5 |
|  | *TC* | 18 | 0 |
|  | *TT* | 36 | 0 |
| g.22304 A>G | *AA* | 34 | 0 |
|  | *AG* | 20 | 0 |
|  | *GG* | 0 | 5 |
| g.23783 G >A | *GG* | 36 | 0 |
|  | *GA* | 18 | 0 |
|  | *AA* | 0 | 5 |
| g.28784 T>C^a^ | *CC* | 0 | 5 |
|  | *TC* | 20 | 0 |
|  | *TT* | 34 | 0 |
| g.32447 A>T^a^ | *AA* | 36 | 0 |
|  | *AT* | 18 | 0 |
|  | *TT* | 0 | 5 |

^a^ These SNPs are synonymous mutation in the coding region of *MUC13B*; the others in the table are intronic polymorphisms. The positions of SNPs refer to GenBank accession no. JN613418.
