# Supplementary material for: Susceptibility towards Enterotoxigenic Escherichia coli F4ac Diarrhea Is Governed by the MUC13 Gene in Pigs
Source: PLoS One. 2012 Sep 12;7(9):e44573. doi: 10.1371/journal.pone.0044573 (PMC3440394; doi:10.1371/journal.pone.0044573)
Supplement: Table S2 — Primers for identification of SNP markers in the region of F4acR that were genotyped in the intercross population. (DOC) [file pone.0044573.s005.doc]

**Supplementary Table 2. Primers for identification of SNP markers in the region of F4acR that were genotyped in the intercross population.**

| Primer pair | Forward primer  (5’-3’) | Reverse primer  (5’-3’) | Tm  (^o^C) | Amplicon  (bp) |
| --- | --- | --- | --- | --- |
| F44/R44 | GTCCTTACCTACACCCCTCAC | TGCCTCCCTACCACACATACC | 60 | 1109 |
| F45/ R45 | CAGTAGAGGAGTTAGAGAGACCCG | CAGAGCCACAGCAACGCAG | 61 | 1348 |
| F48/ R48 | CAGGATGCCCAATGGCTCTAC | CCCCGAAGTTGTGAAAGGAAG | 65 | 538 |
| TNK2-F/TNK2-R | TCAGACCCCAAGTACGCCAC | TTCCAGTCGAACATCTCCAGC | 62 | 1457 |
| TNK2-2F/TNK2-2R | GTGGCTGTGAAGTGCCTGAAG | CACCTGCACAGCGTAGCGG | 59 | 347 |
| B3GnT5b-F/B3GnT5b-R | CGGCAATATAACTTACCTACTTC | TCCCCTAGCAATAGACATCAC | 54 | 1040 |
| B3GnT5A-F/B3GnT5A-R | CATCAGAATGTTTGGGAGTGG | CGTAGGTAAGTTATATTGAAGGGC | 57 | 1320 |
| B3GnT5B-F/B3GnT5B-R | TTCACTGTCACTGAGCCAATC | TCCCCTAGCAATAGACATCAC | 56 | 1176 |
| B3GALT3a-F/B3GALT3a-R | CCTTCCGAGTAAGATGTCCCTG | TGGCATGTGGTGTTCCTTAGC | 61 | 966 |
| MUC13-F/MUC13-R | TGAGCAAGATGAGTGCCCCAGT | TAGCCAGGCAGGCACAAGCA | 67 | 536 |
| M20E-F/M20E-R | GAGACACCAGCCACCCGTAG | TGTGTCTTCATTGGAGTCAGGC | 67 | 430 |
| TFRC-2F/TFRC-2R | TGTCTGCTATGGGATTATTGC | TCTGCTTCGAAAGTTTCTGTC | 57 | 650 |
| MYLK-F/MYLK-R | CGCATCATTGACGAGGACTTTG | GAGGACCTTCAGAGACCCCGC | 64 | 1200 |
| KPNA1-F/KPNA1-R | TGGAGAACAAGAAGCCAAAAG | CCCGAAGTAATGCTCAATAAG | 61 | 2500 |
