# Supplementary material for: Susceptibility towards Enterotoxigenic Escherichia coli F4ac Diarrhea Is Governed by the MUC13 Gene in Pigs
Source: PLoS One. 2012 Sep 12;7(9):e44573. doi: 10.1371/journal.pone.0044573 (PMC3440394; doi:10.1371/journal.pone.0044573)
Supplement: Table S3 — The microsatellite and SNP markers in the region of F4acR that were genotyped in the intercross population. (DOC) [file pone.0044573.s006.doc]

**Supplementary Table 3.** The microsatellite and SNP markers in the region of F4acR that were genotyped in the intercross population.

| No | Polymorphism | Pos. on SSC13  (Sscrofa10.2, bp)^b^ | Forward primer  (5’-3’) | Reverse primer  (5’-3’) | Amplicon  (bp) | Tm  (^o^C) |
| --- | --- | --- | --- | --- | --- | --- |
|  | S0282 | 3457663 | AACTTCCATATGCCACAGGTGC | AGTGGAACAGAATGGAGAGCCC | 103-134 | 65 |
|  | SW1378 | 12278637 | ACCACACGTCTAATTGAAGAGC | TAAATCACAACTTTTGGGGATG | 86-106 | 58 |
|  | SW864 | 34148711 | TTGCACAGATGCTAATTCTTCC | TTAAGACTGTCTTGGGCATTCC | 167-183 | 55 |
|  | SWR2054 ^a^ | 45061916 | TTTGGAGTTAACTGTTGGTGG | AAAGCCCACAACACTGTAAATC | 94-107 | 57 |
|  | SW1495 | 89723347 | AGACGCTGCTTGGTGTTAGG | TGGGTCTGTATCCCTGAAGG | 142-173 | 58 |
|  | SW1105 ^a^ | 109338454 | TTCAATTCAAAGAAGTGTTTGTG | GGTCGATGATGCTCACACC | 270-303 | 58 |
|  | B3GALT3 A>G | 107008228 | CCTTCCGAGTAAGATGCTCCTG | TGGCATGTGGTGTTCCTTAGC | 966 | 50 |
|  | S0084 ^a^ | 116312779 | TGCTGGGCTGTACCAACTC | CCTTTCTCTTTGTTCCATTTC | 99-119 | 56 |
|  | SW1979 | 125989372 | TCCCCTTGCTGTATAGTGGG | ACTGGCTTTGTTTTACACATGG | 140-172 | 63 |
|  | B3GNT5 A>G | 127836935 | GCGAATATGAAGAACAAAGAAATG | TCAGTTAGGGCAGAGCCAAAG | 500 | 63 |
|  | B3GNT5 G>T | 127837082 | GCTTTATGACGGGTTTTTTCG | TGACTTTTCTATCACTTTCATTCTGC | 454 | 56 |
|  | B3GNT5 T>C | 127837322 | CGAAAAAACCCGTCATAAAG | GTTATTAAGGTGCCTTGGTG | 313 | 54 |
|  | SW163 ^a^ | 128084517 | CGCAAATTTAGCTATCCCTGAG | GCTCAGCCCTTTCCCCTAC | 166-187 | 60 |
|  | SW207 | 129685634 | CGCTTCACAAAATAAGTTGGG | GTTGTTACTCCCAAAAAGGTGC | 174-193 | 58 |
|  | UMNp358 ^a^ | 132211001 | AAGTCATTTCACACCTCTGTGC | CGTTGCAGTTACTATTCCAAGC | 131-158 | 56 |
|  | UMNp997 ^a^ | 137096609 | CGGGCAGTGTGTGTCAAG | CACAGACGCATGTGTACAC | 238-254 | 61 |
|  | UMNp595 ^a^ | 139292539 | GGAACTTTCATATGCTGCAGC | CGTTTTTTTTTCCTCCTCAGG | 125-142 | 65 |
|  | TFRC A>G ^a^ | 139706717 | TGTCTGCTATGGGATTATTGC | TCTGCTTCGAAAGTTTCTGTC | 650 | 57 |
|  | TNK2 G>A ^a^ | 139838412 | TGAAGTGCCTGAAGCCCGATG | CAGCCAGTGAACACAAGCCAAAC | 361 | 66 |
|  | TNK2 C>T | 139851249 | TGCAGCTCTCCTCCCACTCAC | CCGCAGACCCAACCCAAATAG | 148 | 59 |
|  | SC1301 ^a^ | 139957152 | AGTTTGTGATGTAGGGGGCG | GCTACAGTTTTGATGAGGTTTGG | 191-302 | 53 |
|  | MUC4 C>T ^a^ | 139960284 | GAAACATAGGATTAGGGTCTTGG | TGGATTGTGTCATTGGGCTC | 354 | 61 |
|  | MUC4 A>G ^a^ | 139962496 | CAGGATGCCCAATGGCTCTAC | CCCCGAAGTTGTGAAAGGAAG | 538 | 56 |
|  | MUC4 C>T ^a^ | 139968506 | AGAACCTCCTCCTGCCAAG | CGGTGATACTGACAGAAGACAAG | 326 | 61 |
|  | MUC4 T>G ^a^ | 139972393 | TCCAACTGCTGCTTTCCTGAC | GTGGGCTATTCCTTTCCTTCG | 360 | 52 |
|  | SC1302 ^a^ | 139980607 | CTTCCACTAACTTTGGTCGTC | ACATGTTCATACAGTGCTGATTAC | 184-190 | 53 |
|  | MUC20 G>A | 139983757 | AAGTGCCACCTGCTCCTTTTGC | GTTCAGGTCACAGGCAGCGATG | 163 | 65 |
|  | MUC20 A>G ^a^ | 139984269 | CGTGATAATCCAAGAGGCAAGTG | CAACAAGAACTGAGACCAGCACC | 175 | 64 |
|  | SC1303 ^a^ | 139984505 | CTTCCAAGGACTCATACCATTC | TAGGAGCGGCACTAGGAAAG | 182-201 | 57 |
|  | SC1304 | 141129977 | TCTAATCAGCACTGCAAAGCA | CTGAGCCATGATGGGAACTC | 176-194 | 65 |
|  | MUC13 T>C | 141131070 | AGAGGCAAAATGAGCAAGATGAG | TTCTAATAGTCAGGGCGGGGTA | 722 | 57 |
|  | MUC13 A>G ^a^ | 141138647 | TTGCCTGTGTTTAGAAGGGTATTACTA | TTCTAATAGTCAGGGCGGGGTA | 176 | 59 |
|  | MUC13 G>C | 141138674 | TTGCCTGTGTTTAGAAGGGTATTACTA | TTCTAATAGTCAGGGCGGGGTA | 176 | 59 |
|  | SWR2189 | - | TATGGCATCCCACAGATTTTG | GCAGCTACAGCTCCAGTTTGA | 154-176 | 61 |
|  | S0283 ^a^ | 141091263 | AGCAGCCCTAGAAATAGCAAGCGC | CTCTCTGTTCCTGGCACCTGGG | 127-141 | 63 |
|  | SW2430 | 142346444 | TGAGAGGAATGAACACTGCTG | GAAGAGCCCTTTTAGGGCC | 129-173 | 60 |
|  | MYLK A>G ^a^ | 142580018 | CAGTTGGTGTTCCAGGCATTG | CTCATTTGCTCCCCCAACCAC | 1200 | 59 |
|  | S0075 ^a^ | 142780951 | GGATCCAAGTGCCAGCAATG | TTGTCCACCCTGGGAGGG | 133-158 | 62 |
|  | KPNA1 A>C ^a^ | 144067920 | TGGTCAGGCGGTTTTGCTTTG | CGCTTGGCAACATTGCTGGAG | 2500 | 65 |
|  | SW482 ^a^ | 153538310 | GCGGAGAAAAGAATGATTATGC | GTCGGTTCTTTGTACCACTCAG | 251-274 | 58 |
|  | SW225 | 155413446 | AGGACCCACCAAGAGTTACC | TGCTGGTAATGGGTGATTAGG | 86-102 | 52 |
|  | SW1386 | 187937911 | GCTGCTTTAGATGGAAAAACTTG | AATAATGAAAGGACTCCTCCAGG | 100-117 | 52 |
|  | SW398 | 189463169 | AAGTGCCAATGCTTTGTTCC | CGGAGGAGAAATAAGGGTAGC | 164-187 | 56 |
|  | SW1056 | 200321969 | GGTGGTTGGTTCTCAAAAACA | TTTCTGGTGTACAGCAAAGTGA | 153-181 | 55 |
|  | SW2440 | 201173028 | CTGGTGTGAAACCCAATCG | CCCCCATAAGGACAGCAATC | 146-160 | 60 |
|  | SW38 | 201186780 | ACGTCTGTGTCGGTGCCT | GAGGCTCCTGATAGCAGCC | 126-137 | 62 |
|  | S0289 | 204210470 | AGGAGCATTTGGCCACGTCTG | TGTTGACCTTCTGTGATGGGGC | 124-183 | 57 |
|  | SW769 | 206464059 | GGTATGACCAAAAGTCCTGGG | TCTGCTATGTGGGAAGAATGC | 104-139 | 56 |
|  | S0215 | 207159068 | TAGGCTCAGACCCTGCTGCAT | TGGGAGGCTGAAGGATTGGGT | 134-200 | 64 |
|  | S0291 | 210277168 | GGAGGGACCCATCTGACAGGA | TTTTGGTGGGACGCTCCTGAC | 148-182 | 56 |

^a^ These markers were genotyped on both F_2_ and F_3_ animals in the White Duroc × Erhualian intercross population.

^b^ The positions of microsatellite markers were determined according to the first nucleotide of forward primers. *SWR2189* that can not accurately map to the pig genome assembly (Sscrofa10) are indicated by ‘-’.
