# Supplementary material for: Susceptibility towards Enterotoxigenic Escherichia coli F4ac Diarrhea Is Governed by the MUC13 Gene in Pigs
Source: PLoS One. 2012 Sep 12;7(9):e44573. doi: 10.1371/journal.pone.0044573 (PMC3440394; doi:10.1371/journal.pone.0044573)
Supplement: Table S4 — Primers for identification of SNP markers in the region of F4acR that were genotyped in outbred populations. (DOC) [file pone.0044573.s007.doc]

**Supplementary Table 4.** Primers for identification of SNP markers in the region of F4acR that were genotyped in outbred populations ^1^.

| No. | Forwardprimer  (5’-3’) | Reverseprimer  (5’-3’) | Tm  (^o^C) | Amplicon  (bp) | Gene | SNP | Pos. on SSC13  (Sscrofa10.2, bp) |
| --- | --- | --- | --- | --- | --- | --- | --- |
|  |  |  |  |  | *GP5* | C>G ^2,3^ | 137340523 |
|  | CAGAAATGATCACAGCCTTGC | GCCACCATATTTCTCTCTACAACAG | 60 | 675 | Intergenic region | C>T ^2^ | 137303607 |
|  |  |  |  |  | *ACAP2* | A>G ^3^ | 138324692 |
|  |  |  |  |  | *APOD* | A>G ^2,3^ | 138612236 |
|  | TTCCACAACAGAAAAACGACAG | TCCTTGTAAATCCTCTTCCGC | 62 | 1300 | *SENP5* | G>A | 139109820 |
|  |  |  |  |  | *PCYT1A* | G>A ^3^ | 139546875 |
|  |  |  |  |  | *OST alpha* | C>T ^3^ | 139597349 |
|  |  |  |  |  | *ZDHHC19* | C>T ^3^ | 139613573 |
|  | ACTTGGCCTATTGTAAACGTG | AGCATTGCTTCCTTTGACTTG | 53 | 1500 | *TFRC* | A>G | 139706717 |
|  | ACTTGGCCTATTGTAAACGTG | AGCATTGCTTCCTTTGACTTG | 53 | 1500 | *TFRC* | G>T | 139717632 |
|  | TCAGACCCCAAGTACGCCAC | TTCCAGTCGAACATCTCCAGC | 62 | 1457 | *TNK2* | G>A | 139838412 |
|  |  |  |  |  | *TNK2* | G>A^3^ | 139846471 |
|  | GCTCACCTATTCCCACAAACG | CCTCTTCATAGGGGATGT | 60 | 1288 | *MUC4* | A>C | 139889433 |
|  | GCTCACCTATTCCCACAAACG | CCTCTTCATAGGGGATGT | 60 | 1288 | *MUC4* | G>A | 139889619 |
|  | GCTCACCTATTCCCACAAACG | CCTCTTCATAGGGGATGT | 60 | 1288 | *MUC4* | T>C | 139889935 |
|  | GGGATTAGGGGAAACCTTGGC | TCCCAACCCTGAAACCACCTC | 63 | 1404 | *MUC4* | T>C | 139899309 |
|  | CAGTTGGGCAGAATGTTTATGAC | GCACGCTGATGCTACTACCG | 59 | 1394 | *MUC4* | C>T | 139903838 |
|  | CAGTTGGGCAGAATGTTTATGAC | GCACGCTGATGCTACTACCG | 59 | 1394 | *MUC4* | T>A | 139904703 |
|  | CCAGAAGCAGATAAGTGAGAAC | GATACCAGATGTGGAAGGATG | 54 | 1315 | *MUC4* | C>T | 139909358 |
|  | CCAGAAGCAGATAAGTGAGAAC | GATACCAGATGTGGAAGGATG | 54 | 1315 | *MUC4* | A>T | 139909613 |
|  | AATGCCTCCTGGATTACTTTC | CTGGAATCTAATACACGGCAC | 55 | 1345 | *MUC4* | A>C | 139915112 |
|  | CAGCAAGAGCCCTGACAAGCC | GCTGAAGGGAACTGAGGTGGATAG | 64 | 1380 | *MUC4* | C>T | 139919669 |
|  | CCAGCAAAGACTTGGCACCTAC | GCAAATACGGATGCCCAGTTC | 62 | 1422 | *MUC4* | A>G | 139923889 |
|  | CCAGCAAAGACTTGGCACCTAC | GCAAATACGGATGCCCAGTTC | 62 | 1422 | *MUC4* | G>A | 139924303 |
|  | CACAGCGGTAGTGAAGAAATC | CTGCTTCTATCTTCTGGCTCTATG | 59 | 1422 | *MUC4* | G>A | 139929524 |
|  | CACAGCGGTAGTGAAGAAATC | CTGCTTCTATCTTCTGGCTCTATG | 59 | 1422 | *MUC4* | T>A | 139929925 |
|  | CAACGGAGAGGGTATTGGGAAGC | AGCCTACGCCAGAGCCACAGC | 66 | 1117 | *MUC4* | A>G | 139932684 |
|  | CAACGGAGAGGGTATTGGGAAGC | AGCCTACGCCAGAGCCACAGC | 66 | 1117 | *MUC4* | T>C | 139933273 |
|  | GCTTAGACAGTGAGACATCAACATC | AATTACAGGTGACACGCTTCC | 57 | 1366 | *MUC4* | C>T | 139937594 |
|  | TTCCAGGAGTTTGATTGTGTC | GTCATAGTGTTTCCACCTGTAGAG | 55 | 1159 | *MUC4* | A>G | 139941078 |
|  | TTCCAGGAGTTTGATTGTGTC | GTCATAGTGTTTCCACCTGTAGAG | 55 | 1159 | *MUC4* | A>C | 139941488 |
|  | CAGAGAAACCCACAGCAGCG | GGGTAAAGAGGATGGTGAGGC | 62 | 1800 | *MUC4* | T>C | 139949309 |
|  | CAGAGAAACCCACAGCAGCG | GGGTAAAGAGGATGGTGAGGC | 62 | 1800 | *MUC4* | C>G | 139949327 |
|  | ACCCTCTTCCACCACCTCTG | GAATGTCCTCTCTCAAGCACTC | 61 | 508 | *MUC4* | C>T | 139949475 |
|  | CAGAGAAACCCACAGCAGCG | GGGTAAAGAGGATGGTGAGGC | 62 | 1800 | *MUC4* | G>C | 139949522 |
|  | GACATCATCAGTGTTGGGTGC | TCAATCACAGTCACAGTTATGGC | 58 | 1339 | *MUC4* | A>G | 139950389 |
|  | GACATCATCAGTGTTGGGTGC | TCAATCACAGTCACAGTTATGGC | 58 | 1339 | *MUC4* | G>A | 139951081 |
|  | AAAAGGGTGACTCAGCAATGG | ACAAGAAGGCAGATTCAGACAAG | 59 | 1295 | *MUC4* | A>G | 139952761 |
|  | CCAGCAAGGACAGACAGAGG | CCACCTGTGCCCATCTGTATC | 58 | 420 | *MUC4* | C>T | 139952774 |
|  | TTGTCACTGCTGTGGCTCTG | CCTGGCTATTTTCTTTCTATTTC | 56 | 121 | *MUC4* | T>C | 139953757 |
|  | TTCACTCTGCCGTTCTCTTTC | CAGCTGTTCAGTCGTTTCGTC | 60 | 1161 | *MUC4* | C>G | 139955122 |
|  | TTCACTCTGCCGTTCTCTTTC | CAGCTGTTCAGTCGTTTCGTC | 60 | 1161 | *MUC4* | G>A | 139955389 |
|  | TTCACTCTGCCGTTCTCTTTC | CAGCTGTTCAGTCGTTTCGTC | 60 | 1161 | *MUC4* | G>A | 139955731 |
|  | GACATCTATTCAGTCTTCGTTTATTG | AAGTCCCATAGTCCCTGCTGC | 59 | 1188 | *MUC4* | A>G | 139957452 |
|  | GACATCTATTCAGTCTTCGTTTATTG | AAGTCCCATAGTCCCTGCTGC | 59 | 1188 | *MUC4* | T>G | 139957661 |
|  | TGTCCACAGAGAACTTGAGCC | GTTTGCCATCTCCACACACTG | 58 | 1306 | *MUC4* | C>T | 139958638 |
|  | TGTCCACAGAGAACTTGAGCC | GTTTGCCATCTCCACACACTG | 58 | 1306 | *MUC4* | T>C | 139958889 |
|  | TGTCCACAGAGAACTTGAGCC | GTTTGCCATCTCCACACACTG | 58 | 1306 | *MUC4* | A>G | 139959469 |
|  | GAAACATAGGATTAGGGTCTTGG | TGGATTGTGTCATTGGGCTC | 58 | 354 | *MUC4* | C>T | 139960284 |
|  | CTCTCAGCCTCCTCTCCACATC | AAGTCATCATCTGGGTTGCCG | 61 | 1365 | *MUC4* | G>C | 139961567 |
|  | CAGGATGCCCAATGGCTCTAC | CCCCGAAGTTGTGAAAGGAAG | 65 | 538 | *MUC4* | A>G | 139962496 |
|  | GTGCCTTTCTTGGCTAACTTGTC | TGTGGCTGTGGTGTAGGTTGG | 61 | 1218 | *MUC4* | C>T | 139964493 |
|  | TGGGACGTTGCTATGGACACC | CTCTACTGAGACGGCTCTGGAAAG | 62 | 1447 | *MUC4* | T>C | 139966387 |
|  | TGGGACGTTGCTATGGACACC | CTCTACTGAGACGGCTCTGGAAAG | 62 | 1447 | *MUC4* | G>A | 139966798 |
|  | CAGTAGAGGAGTTAGAGAGACCCG | CAGAGCCACAGCAACGCAG | 61 | 1348 | *MUC4* | C>T | 139968506 |
|  | CCTTGGCTAAGTCTGGGTAAAC | AAATGAACAGCCCCTACCTTG | 58 | 1094 | *MUC4* | C>T | 139970271 |
|  | GTCCTTACCTACACCCCTCAC | TGCCTCCCTACCACACATACC | 60 | 1109 | *MUC4* | T>G | 139972393 |
|  | GTGTAGTCACTGTAGAGGCTTAGG | CTATCCATTTGCTGGTTTGAG | 55 | 1432 | *MUC4* | A>G | 139974210 |
|  | GTGTAGTCACTGTAGAGGCTTAGG | CTATCCATTTGCTGGTTTGAG | 55 | 1432 | *MUC4* | G>A | 139974975 |
|  | GTGTAGTCACTGTAGAGGCTTAGG | CTATCCATTTGCTGGTTTGAG | 55 | 1432 | *MUC4* | T>C | 139975219 |
|  | GCCCTACTACAGTCGCAGATG | GGGGTTACTGGGAGATGGTTG | 59 | 1326 | *MUC4* | G>A | 139975952 |
|  | GCCCTACTACAGTCGCAGATG | GGGGTTACTGGGAGATGGTTG | 59 | 1326 | *MUC4* | G>A | 139976758 |
|  | GCCCATTATCTAAGTCATTCAGC | AGTATCCTCCCAGCAGGTCTC | 58 | 1334 | *MUC4* | C>T | 139981552 |
|  | GCCCATTATCTAAGTCATTCAGC | AGTATCCTCCCAGCAGGTCTC | 58 | 1334 | *MUC4* | C>G | 139981718 |
|  | GCCCATTATCTAAGTCATTCAGC | AGTATCCTCCCAGCAGGTCTC | 58 | 1334 | *MUC4* | G>A | 139981881 |
|  | CCAGAAGACCTCACTGACCCCACA | GTTCAGGTCACAGGCAGCGATG | 71 | 731 | *MUC20* | G>A | 139983757 |
|  | CCAGAAGACCTCACTGACCCCACA | GTTCAGGTCACAGGCAGCGATG | 71 | 731 | *MUC20* | C>T | 139984374 |
|  | ATAAGACCAGACCCTCCCAAG | CTGAAACTCACAGTCTCCCATTG | 58 | 1474 | *MUC20* | C>A | 139986099 |
|  | ATAAGACCAGACCCTCCCAAG | CTGAAACTCACAGTCTCCCATTG | 58 | 1474 | *MUC20* | G>C | 139986930 |
|  | GAGACACCAGCCACCCGTAG | TGTGTCTTCATTGGAGTCAGGC | 67 | 430 | *MUC20* | T>C | 139989479 |
|  | GGGAGGGTTGCCAGATTTAGC | CAAGATGACAACACGGGGCAC | 63 | 1268 | *MUC20* | G>A | 139991264 |
|  | GGGAGGGTTGCCAGATTTAGC | CAAGATGACAACACGGGGCAC | 63 | 1268 | *MUC20* | A>G | 139991440 |
|  | GGGAGGGTTGCCAGATTTAGC | CAAGATGACAACACGGGGCAC | 63 | 1268 | *MUC20* | T>C | 139992083 |
|  | GTGTGAGGCTTCGCCCTTGAG | CAGAATGTGACCAACTCCCACTACC | 64 | 1192 | Intergenic region | C>T | 139997118 |
|  | AAGACAGGCAGGGACAGTTGG | GTTGTTGTTGTTGAGTTTGTGAGAG | 60 | 1255 | Intergenic region | T>C | 140000949 |
|  | AAGACAGGCAGGGACAGTTGG | GTTGTTGTTGTTGAGTTTGTGAGAG | 60 | 1255 | Intergenic region | C>T | 140001250 |
|  | TCTAAGCACCCAGCATACTCC | CTCTTCCTTCTTTTCATACCATC | 56 | 1195 | Intergenic region | G>A | 140006421 |
|  | GGCAACAGGGAAGACCGTATC | CTATGTCATTGCTTCCAACCTTC | 60 | 1190 | Intergenic region | A>G | 140010850 |
|  | GGCAACAGGGAAGACCGTATC | CTATGTCATTGCTTCCAACCTTC | 60 | 1190 | Intergenic region | G>A | 140011540 |
|  | GTCACTGCTATGATGAAAGGTC | GCAGATGAATGGATTGAGAAG | 54 | 1301 | Intergenic region | G>A | 140016672 |
|  | GTCACTGCTATGATGAAAGGTC | GCAGATGAATGGATTGAGAAG | 54 | 1301 | Intergenic region | G>A | 140031136 |
|  | GCCTTTAGAACGAACTATCAC | AGGTACTTCTGTATGAAACGC | 60 | 1500 | *ZNF148* | A>G | 140675072 |
|  | ACCAACTTCTTTCTACACCCG | GAAATACACAATGGCAGCAAC | 58 | 750 | *SLC12A8* | G>A | 140947266 |
|  | GGTTTCCACAGTTGAGWCCAC | CATGTGAAGATGGATATAGGC | 60 | 200 | *HEG1* | A>G | 140768319 |
|  | TGTGTCTGTGGGAGGAGGTG | CTGTGAGCTGTGGTGTAGGC | 58 | 1379 | Intergenic region | T>C | 141002191 |
|  | AAGCAAGTCAGCCAGTCAAGG | CAAACTCTCCATCCATCCCAC | 59 | 1420 | Intergenic region | T>C | 141006285 |
|  | AAGCAAGTCAGCCAGTCAAGG | CAAACTCTCCATCCATCCCAC | 59 | 1420 | Intergenic region | C>T | 141006611 |
|  | CAGAACCCTCATTTCATCACC | GAGATAAACGAGAAGGTCCTACTG | 57 | 1350 | Intergenic region | T>C | 141011451 |
|  | CAGAACCCTCATTTCATCACC | GAGATAAACGAGAAGGTCCTACTG | 57 | 1350 | Intergenic region | C>T | 141012345 |
|  | GTTAGAAAGACTGATGATGTGGAG | GTTTTGAGGAGAGAAGTTGAGC | 55 | 1240 | Intergenic region | G>A | 141018068 |
|  | GTTAGAAAGACTGATGATGTGGAG | GTTTTGAGGAGAGAAGTTGAGC | 55 | 1240 | Intergenic region | G>A | 141018325 |
|  | GTTAGAAAGACTGATGATGTGGAG | GTTTTGAGGAGAGAAGTTGAGC | 55 | 1240 | Intergenic region | G>C | 141018578 |
|  | GGGAAGTGATAGAAGCATTGTG | CTTTGTCGTCTTTCGCTGTG | 57 | 1370 | Intergenic region | T>C | 141019044 |
|  | GGGAAGTGATAGAAGCATTGTG | CTTTGTCGTCTTTCGCTGTG | 57 | 1370 | Intergenic region | T>G | 141019113 |
|  | GGGAAGTGATAGAAGCATTGTG | CTTTGTCGTCTTTCGCTGTG | 57 | 1370 | Intergenic region | A>G | 141019392 |
|  | GGGAAGTGATAGAAGCATTGTG | CTTTGTCGTCTTTCGCTGTG | 57 | 1370 | Intergenic region | T>A | 141019747 |
|  | GGGAAGTGATAGAAGCATTGTG | CTTTGTCGTCTTTCGCTGTG | 57 | 1370 | Intergenic region | C>A | 141020219 |
|  | GACAAAGTTGCTAAAAGTGACC | GTAGTGAGACTCTAAGGCATTGG | 55 | 1423 | Intergenic region | C>T | 141020585 |
|  | GACAAAGTTGCTAAAAGTGACC | GTAGTGAGACTCTAAGGCATTGG | 55 | 1423 | Intergenic region | T>C | 141020904 |
|  | AGATATCACTTGGGTTCTAATGC | CACAAGGTGGCACTGTTACTAC | 55 | 1423 | *MUC13* | C>G | 141022725 |
|  | AGATATCACTTGGGTTCTAATGC | CACAAGGTGGCACTGTTACTAC | 55 | 1423 | *MUC13* | A>T | 141022976 |
|  | TACACCTATTGCCTCCACACC | AGAACACAAGAAGACTGCCTTACTC | 58 | 1274 | *MUC13* | C>T | 141190628 |
|  | GCGTTAGGGTTTACAGTTGATG | ATTTGTCTGTCTTCCCTTTGG | 57 | 1427 | *MUC13* | G>A | 141191837 |
|  | CATATGTGGTGGGGAAAGGC | AGTGTTCCCTAAAACCATTTCAGAG | 60 | 1161 | *MUC13* | G>T | 141193063 |
|  | TCCGACTAGGAACCATGAGG | TTTTCATGTTCTAGATCAGTTG | 60 | 1792 | *MUC13* | A>G | 141121173 |
|  | TCCGACTAGGAACCATGAGG | TTTTCATGTTCTAGATCAGTTG | 60 | 1792 | *MUC13* | A>T | 141121197 |
|  | TCCGACTAGGAACCATGAGG | TTTTCATGTTCTAGATCAGTTG | 60 | 1792 | *MUC13* | G>A | 141121229 |
|  | TCCGACTAGGAACCATGAGG | TTTTCATGTTCTAGATCAGTTG | 60 | 1792 | *MUC13* | G>A | 141121280 |
|  | TCCGACTAGGAACCATGAGG | TTTTCATGTTCTAGATCAGTTG | 60 | 1792 | *MUC13* | G>A | 141121430 |
|  | TCCGACTAGGAACCATGAGG | TTTTCATGTTCTAGATCAGTTG | 60 | 1792 | *MUC13* | G>A | 141121431 |
|  | TCCGACTAGGAACCATGAGG | TTTTCATGTTCTAGATCAGTTG | 60 | 1792 | *MUC13* | G>T | 141121479 |
|  | TCCGACTAGGAACCATGAGG | TTTTCATGTTCTAGATCAGTTG | 60 | 1792 | *MUC13* | G>A | 141121516 |
|  | TCCGACTAGGAACCATGAGG | TTTTCATGTTCTAGATCAGTTG | 60 | 1792 | *MUC13* | T>C | 141121531 |
|  | TCCGACTAGGAACCATGAGG | TTTTCATGTTCTAGATCAGTTG | 60 | 1792 | *MUC13* | A>G | 141121540 |
|  | TCCGACTAGGAACCATGAGG | TTTTCATGTTCTAGATCAGTTG | 60 | 1792 | *MUC13* | G>A | 141121561 |
|  | TCCGACTAGGAACCATGAGG | TTTTCATGTTCTAGATCAGTTG | 60 | 1792 | *MUC13* | G>A | 141121597 |
|  | TCCGACTAGGAACCATGAGG | TTTTCATGTTCTAGATCAGTTG | 60 | 1792 | *MUC13* | A>G | 141121702 |
|  | TCCGACTAGGAACCATGAGG | TTTTCATGTTCTAGATCAGTTG | 60 | 1792 | *MUC13* | C>T | 141121745 |
|  | TCCGACTAGGAACCATGAGG | TTTTCATGTTCTAGATCAGTTG | 60 | 1792 | *MUC13* | T>C | 141121774 |
|  | TCCGACTAGGAACCATGAGG | TTTTCATGTTCTAGATCAGTTG | 60 | 1792 | *MUC13* | G>A | 141121787 |
|  | TCCGACTAGGAACCATGAGG | TTTTCATGTTCTAGATCAGTTG | 60 | 1792 | *MUC13* | G>A | 141121799 |
|  | TCCGACTAGGAACCATGAGG | TTTTCATGTTCTAGATCAGTTG | 60 | 1792 | *MUC13* | A>G | 141121812 |
|  | TCCGACTAGGAACCATGAGG | TTTTCATGTTCTAGATCAGTTG | 60 | 1792 | *MUC13* | A>G | 141121844 |
|  | TCCGACTAGGAACCATGAGG | TTTTCATGTTCTAGATCAGTTG | 60 | 1792 | *MUC13* | C>T | 141121887 |
|  | TCCGACTAGGAACCATGAGG | TTTTCATGTTCTAGATCAGTTG | 60 | 1792 | *MUC13* | G>A | 141122000 |
|  | TCCGACTAGGAACCATGAGG | TTTTCATGTTCTAGATCAGTTG | 60 | 1792 | *MUC13* | A>G | 141122022 |
|  | TCCGACTAGGAACCATGAGG | TTTTCATGTTCTAGATCAGTTG | 60 | 1792 | *MUC13* | G>A | 141122101 |
|  | TCCGACTAGGAACCATGAGG | TTTTCATGTTCTAGATCAGTTG | 60 | 1792 | *MUC13* | C>A | 141122122 |
|  | TCCGACTAGGAACCATGAGG | TTTTCATGTTCTAGATCAGTTG | 60 | 1792 | *MUC13* | C> A | 141122128 |
|  | AGCTTTCCATTTCTTCACATCTT | GAAATGCCTCTCTTCACATTAAAT | 60 | 739 | *MUC13* | A>G | 141122318 |
|  | AGCTTTCCATTTCTTCACATCTT | GAAATGCCTCTCTTCACATTAAAT | 60 | 739 | *MUC13* | C>T | 141122462 |
|  | CACTGAAAACAAGAAATGACTGC | GTAGATTGCTTTGGGTAGTATGG | 57 | 1284 | *MUC13* | T>C | 141122511 |
|  | AGCTTTCCATTTCTTCACATCTT | GAAATGCCTCTCTTCACATTAAAT | 60 | 739 | *MUC13* | C>T | 141122697 |
|  | CCTTTTTCTCCACACCCTCTCC | AAACTGGGAGGTTGCGATTG | 62 | 1463 | *MUC13* | T>C | 141124335 |
|  | GAGATTCTTTCCCCAAATAGGTC | TGGTGTAGGCTGGCAGTGAC | 59 | 1353 | *MUC13* | T>G | 141126211 |
|  | GAGATTCTTTCCCCAAATAGGTC | TGGTGTAGGCTGGCAGTGAC | 59 | 1353 | *MUC13* | G>A | 141126357 |
|  | AGCAATTCTGAGTACGGACAGAC | AAATATGGAACTCTCATTTTCTGTTGT | 62 | 3440 | *MUC13* | G>A | 141128303 |
|  | AGCAATTCTGAGTACGGACAGAC | AAATATGGAACTCTCATTTTCTGTTGT | 62 | 3440 | *MUC13* | G>A | 141128385 |
|  | AGCAATTCTGAGTACGGACAGAC | AAATATGGAACTCTCATTTTCTGTTGT | 62 | 3440 | *MUC13* | G>C | 141128525 |
|  | AGCAATTCTGAGTACGGACAGAC | AAATATGGAACTCTCATTTTCTGTTGT | 62 | 3440 | *MUC13* | C>G | 141130371 |
|  | AGCAATTCTGAGTACGGACAGAC | AAATATGGAACTCTCATTTTCTGTTGT | 62 | 3440 | *MUC13* | G>A | 141130540 |
|  | ACACCTATTGCCTCCACACC | AATCTCATGTGCTGGGTTGAC | 58 | 1464 | *MUC13* | A>G | 141130912 |
|  | ACACCTATTGCCTCCACACC | AATCTCATGTGCTGGGTTGAC | 58 | 1464 | *MUC13* | G>C | 141130985 |
|  | ACACCTATTGCCTCCACACC | AATCTCATGTGCTGGGTTGAC | 58 | 1464 | *MUC13* | C>G | 141130991 |
|  | GTCTCTCCAGCCCCTCTTTG | GAAATGCGTGCCAGCAAC | 63 | 3878 | *MUC13* | C>A | 141131211 |
|  | GTCTCTCCAGCCCCTCTTTG | GAAATGCGTGCCAGCAAC | 63 | 3878 | *MUC13* | C>A | 141131615 |
|  | GTCTCTCCAGCCCCTCTTTG | GAAATGCGTGCCAGCAAC | 63 | 3878 | *MUC13* | G>A | 141131777 |
|  | GTCTCTCCAGCCCCTCTTTG | GAAATGCGTGCCAGCAAC | 63 | 3878 | *MUC13* | G>A | 141133993 |
|  | GTCTCTCCAGCCCCTCTTTG | GAAATGCGTGCCAGCAAC | 63 | 3878 | *MUC13* | T>C | 141134125 |
|  | GTCTCTCCAGCCCCTCTTTG | GAAATGCGTGCCAGCAAC | 63 | 3878 | *MUC13* | A>G | 141134358 |
|  | GTCTCTCCAGCCCCTCTTTG | GAAATGCGTGCCAGCAAC | 63 | 3878 | *MUC13* | G>C | 141134492 |
|  | GTCTCTCCAGCCCCTCTTTG | GAAATGCGTGCCAGCAAC | 63 | 3878 | *MUC13* | C>T | 141134629 |
|  | ACACCTATTGCCTCCACACC | AATCTCATGTGCTGGGTTGAC | 58 | 1464 | *MUC13* | T>C | 141134778 |
|  | TGAGCAAGATGAGTGCCCCAGT | TAGCCAGGCAGGCACAAGCA | 67 | 536 | *MUC13* | G>A | 141134958 |
|  | TGAGCAAGATGAGTGCCCCAGT | TAGCCAGGCAGGCACAAGCA | 67 | 536 | *MUC13* | A>G | 141135046 |
|  | TGAGCAAGATGAGTGCCCCAGT | TAGCCAGGCAGGCACAAGCA | 67 | 536 | *MUC13* | T>C | 141135124 |
|  | ACACCTATTGCCTCCACACC | AATCTCATGTGCTGGGTTGAC | 58 | 1464 | *MUC13* | C>T | 141135254 |
|  | ACACCTATTGCCTCCACACC | AATCTCATGTGCTGGGTTGAC | 58 | 1464 | *MUC13* | T>C | 141135276 |
|  | ACACCTATTGCCTCCACACC | AATCTCATGTGCTGGGTTGAC | 58 | 1464 | *MUC13* | G>A | 141135316 |
|  | CCCATGAACACAGTTTTGGA | AGCAATTGGAACCACAGGAG | 59 | 903 | *MUC13* | G>A | 141136439 |
|  | CATTTGGCTACAGTGGAGTTGG | ACCAGGCTGAGAATGAGGATG | 60 | 1712 | *MUC13* | T>C | 141138096 |
|  | TCGCTGGCATCCTCATTCTC | CTCAATCAAGTTCTGTTCTTCCAC | 61 | 2250 | *MUC13* | G>A | 141138789 |
|  | ATGTGGAAGAACAGAACTTGATTGAG | ATAGTCAGGGCGGGGTATACTACC | 58 | 176 | *MUC13* | C>T | 141141435 |
|  | ATGTGGAAGAACAGAACTTGATTGAG | ATAGTCAGGGCGGGGTATACTACC | 58 | 176 | *MUC13* | T>G | 141141436 |
|  | ATGTGGAAGAACAGAACTTGATTGAG | ATAGTCAGGGCGGGGTATACTACC | 58 | 176 | *MUC13* | A>C | 141141486 |
|  | ATGTGGAAGAACAGAACTTGATTGAG | ATAGTCAGGGCGGGGTATACTACC | 58 | 176 | *MUC13* | C>A | 141141517 |
|  | ATGTGGAAGAACAGAACTTGATTGAG | ATAGTCAGGGCGGGGTATACTACC | 58 | 176 | *MUC13* | A>G | 141141527 |
|  | ATGTGGAAGAACAGAACTTGATTGAG | ATAGTCAGGGCGGGGTATACTACC | 58 | 176 | *MUC13* | A>G | 141141530 |
|  | ATGTGGAAGAACAGAACTTGATTGAG | ATAGTCAGGGCGGGGTATACTACC | 58 | 176 | *MUC13* | G>A | 141141542 |
|  | ATGTGGAAGAACAGAACTTGATTGAG | ATAGTCAGGGCGGGGTATACTACC | 58 | 176 | *MUC13* | T>C | 141141555 |
|  | ATGTGGAAGAACAGAACTTGATTGAG | ATAGTCAGGGCGGGGTATACTACC | 58 | 176 | *MUC13* | A>C | 141141557 |
|  | ACACCTATTGCCTCCACACC | AATCTCATGTGCTGGGTTGAC | 58 | 1464 | *MUC13* | C>A | 141143831 |
|  | AATGGAAGCTGTAACTGTTTGC | GCAAGGAGACCAGACATCAGAC | 58 | 1123 | *MUC13* | G>A | 141144290 |
|  | AATGGAAGCTGTAACTGTTTGC | GCAAGGAGACCAGACATCAGAC | 58 | 1123 | *MUC13* | G>A | 141144430 |
|  | AATGGAAGCTGTAACTGTTTGC | GCAAGGAGACCAGACATCAGAC | 58 | 1123 | *MUC13* | A>T | 141145074 |
|  | GTGGATGGTGACGAGCAGC | GGAGGCTGTGCTTTGTTTCTAC | 62 | 1800 | *ITGB5* | C>T | 141354227 |
|  | GTGAGGGAGCGGTCTGAACG | AATCTCCTCCGTGAAGCAGCC | 67 | 2000 | *ROPN1B* | C>T | 142138794 |
|  | TGTTATCCTCTGACCACGAC | GCACAATCATCTCTGTATCTTC | 57 | 850 | *ROPN1* | A>G | 142138900 |
|  | GAGCCAGGAATTACTGCAGAG | AGTCACCTCAGCATCACGC | 63 | 370 | *CCDC14* | A>G | 142213330 |
|  | CGCATCATTGACGAGGACTTTG | GAGGACCTTCAGAGACCCCGC | 64 | 1200 | *MYLK* | T>C | 142580018 |
|  | TTACATAAAACATCCTGGC | ACACACTGGGGTTGAGAC | 55 | 2100 | *PTPLB* | C>T ^2^ | 142698140 |
|  | ATCTGGGGCAATACSGTGAAC | AGGTCATCATCTCGCCTTTGC | 56 | 2000 | *ADCY5* | G>C ^2^ | 142924627 |
|  | TCCGACTAGGAACCATGAGG | TTTTCATGTTCTAGATCAGTTG | 60 | 1792 | *MUC13* | C>T | - |
|  | TCCGACTAGGAACCATGAGG | TTTTCATGTTCTAGATCAGTTG | 60 | 1792 | *MUC13* | C>T | - |
|  | CTGTGTTTCTAAGACTTGACTG | TGTTCAAGAAGAGCATCAAG | 57 | 600 | *KALRN* | A>T | - |
|  | ACCCTGGCTGCTCGGAATCTG | ACAGGTTCACGGGCAGGTCAC | 65 | 1700 | *PLXNA1* | G>T | - |
|  | AAGCAAATCGGAAGGCTGAAG | CGCACTTTGTTAGTTTGTTGGTAG | 59 | 355 | Intergenic region | C>T | - |
|  | GATACAGGTTCAGTCCTTGCC | TCAAGTGTGATCCAAGAGTTTC | 58 | 520 | Intergenic region | C>T | - |

^1^ Ten SNPs that cannot accurately map to the pig genome assembly (Sscrofa9.2) are indicated by‘-’;

^2^ These SNPs are located outside the 3.1-Mb critical region harboring the receptor locus;

^3^ SNPs from Jacobsen et al.[7].
