# Supplementary material for: Susceptibility towards Enterotoxigenic Escherichia coli F4ac Diarrhea Is Governed by the MUC13 Gene in Pigs
Source: PLoS One. 2012 Sep 12;7(9):e44573. doi: 10.1371/journal.pone.0044573 (PMC3440394; doi:10.1371/journal.pone.0044573)
Supplement: Table S5 — Primers for isolation of the full-length cDNA and genomic DNA sequence of the porcine MUC13 gene. (DOC) [file pone.0044573.s008.doc]

**Supplementary Table 5.** Primers for isolation of the full-length cDNA and genomic DNA sequence of the porcine *MUC13* gene.

| Primer pairs | Primer sequence  (5’-3’) | Amplicon  (bp) | Tm  (^o^C) |
| --- | --- | --- | --- |
| F1/R1 | F: CTACCCAGAGGCTTCCACAC | 244 | 62 |
|  | R: CACAGTGGGAACTTCCAAAG |  |  |
| F2/R2 | F: ACACCCACCTCAGGTAAGAGAG | 635 (*MUC13A*) | 55 |
|  | R: GCAAGTGGTTTTGGTTTCAACT | 577 (*MUC13B*) |  |
| F3/UPM | F: gggttgcttttaattgcttggtcaatg | 630 | 68 |
|  | R: CTAATACGACTCACTATAGGGCAAGCAGTGGTATCAACGCAGAGT  CTAATACGACTCACTATAGGGC |  |  |
| NF3/NUP | F: CATGAAGAACCACCCTTACAGGAATCTTG | 320 (*MUC13A*) | 68 |
|  | R: AAGCAGTGGTATCAACGCAGAGT | 396 (*MUC13B*) |  |
| F4/5’RACE Out Primer | F: GATTTTAGGAAAAGGAGGGCAAGCAGAG | 141 | 55 |
|  | R: CATGGCTACATGCTGACAGCCTA |  |  |
| NF4/5’RACE Inner Primer | F: GGCAAGCAGAGTGAAGTGAATGAAGACT | 110 | 55 |
|  | R: CGCGGATCCACAGCCTACTGATGATCAGTCGATG |  |  |
| F5/5’RACE Out Primer | F: GAGTTGTRGTTGGTGTGGGGGT | 427 | 55 |
|  | R: CATGGCTACATGCTGACAGCCTA |  |  |
| NF5/5’RACE Inner Primer | F: GTTGGTGTGGGGGTRRGAGTTGT | 404 | 55 |
|  | R: CGCGGATCCACAGCCTACTGATGATCAGTCGATG |  |  |
| F6/R6 | F: TTCTCTCTGGATTACCTGGCACCAAC | 168 | 65 |
|  | R: GCTTTCCGCCGCGTTCGGGGAG |  |  |
