# Supplementary material for: Susceptibility towards Enterotoxigenic Escherichia coli F4ac Diarrhea Is Governed by the MUC13 Gene in Pigs
Source: PLoS One. 2012 Sep 12;7(9):e44573. doi: 10.1371/journal.pone.0044573 (PMC3440394; doi:10.1371/journal.pone.0044573)
Supplement: Table S6 — Primers for the copy number assay of the porcine MUC13 gene. (DOC) [file pone.0044573.s009.doc]

**Supplementary Table 6.** Primers for the copy number assay of the porcine *MUC13* gene.

| Primer pairs | Primer sequence  (5’-3’) | Amplicon  (bp) | Tm  (^o^C) |
| --- | --- | --- | --- |
| MUC13-FP1/RP1 | F: AGCAAACTAACCCTAGAGCATCCAGTCCCGTCTTATTTCAGTG | 437 | 60 |
|  | R: TTCCCGTATTTAGGGTCAT |  |  |
| GAPDH-FP1/RP1 | F: GGCTCTTTCTTTCCTTTCGC | 368 | 60 |
|  | R: CACTGAAATAAGACGGGACTGGATGCTCTAGGGTTAGTTTGCT |  |  |
| MUC13-FP/RP/Probe | F: ACCTGACCTGTTCAAGGGAATG | 68 | 60 |
|  | R: TCCAGGGACGTCAACACCTATC |  |  |
|  | TaqMan Probe: CCATGGGATCAAAGTCAGA |  |  |
| GAPDH-FP/RP/Probe | F: ATGAATGAACCGCCGTTAGG | 66 | 60 |
|  | R: AGTCATGGGCAAGTAAGGAGAAAG |  |  |
|  | TaqMan Probe: AATCTTCCTGAGTCCTTCA |  |  |
